# Pyocycanin, a Contributory Factor in Haem Acquisition and Virulence Enhancement of *Porphyromonas gingivalis* in the Lung

**DOI:** 10.1371/journal.pone.0118319

**Published:** 2015-02-23

**Authors:** Malgorzata Benedyk, Dominic P. Byrne, Izabela Glowczyk, Jan Potempa, Mariusz Olczak, Teresa Olczak, John W. Smalley

**Affiliations:** 1 School of Dentistry, University of Liverpool, Liverpool, United Kingdom; 2 Department of Microbiology, Faculty of Biochemistry, Biophysics and Biotechnology, Jagiellonian University, Krakow, Poland; 3 Department of Biochemistry and Cell Biology, Institute of Integrative Biology, University of Liverpool, Liverpool, United Kingdom; 4 Faculty of Biotechnology, Laboratory of Biochemistry, University of Wroclaw, Wroclaw, Poland; 5 Department of Oral Immunology and Infectious Diseases, University of Louisville School of Dentistry, Louisville, United States of America; University of Florida, UNITED STATES

## Abstract

Several recent studies show that the lungs infected with *Pseudomonas aeruginosa* are often co-colonised by oral bacteria including black-pigmenting anaerobic (BPA) *Porphyromonas* species. The BPAs have an absolute haem requirement and their presence in the infected lung indicates that sufficient haem, a virulence up-regulator in BPAs, must be present to support growth. Haemoglobin from micro-bleeds occurring during infection is the most likely source of haem in the lung. *Porphyromonas gingivalis* displays a novel haem acquisition paradigm whereby haemoglobin must be firstly oxidised to methaemoglobin, facilitating haem release, either by gingipain proteolysis or capture *via* the haem-binding haemophore HmuY. *P. aeruginosa* produces the blue phenazine redox compound, pyocyanin. Since phenazines can oxidise haemoglobin, it follows that pyocyanin may also facilitate haem acquisition by promoting methaemoglobin production. Here we show that pyocyanin at concentrations found in the CF lung during *P. aeruginosa* infections rapidly oxidises oxyhaemoglobin in a dose-dependent manner. We demonstrate that methaemoglobin formed by pyocyanin is also susceptible to proteolysis by *P. gingivalis* Kgp gingipain and neutrophil elastase, thus releasing haem. Importantly, co-incubation of oxyhaemoglobin with pyocyanin facilitates haem pickup from the resulting methemoglobin by the *P. gingivalis* HmuY haemophore. Mice intra-tracheally challenged with viable *P. gingivalis* cells plus pyocyanin displayed increased mortality compared to those administered *P. gingivalis* alone. Pyocyanin significantly elevated both methaemoglobin and total haem levels in homogenates of mouse lungs and increased the level of arginine-specific gingipain activity from mice inoculated with viable *P. gingivalis* cells plus pyocyanin compared with mice inoculated with *P. gingivalis* only. These findings indicate that pyocyanin, by promoting haem availability through methaemoglobin formation and stimulating of gingipain production, may contribute to virulence of *P. gingivalis* and disease severity when co-infecting with *P. aeruginosa* in the lung.

## Introduction

*Pseudomonas aeruginosa* is one of the major bacterial causes of respiratory infections- in patients with cystic fibrosis (CF), and contributes to acute exacerbations of chronic obstructive pulmonary disease, or bronchiectasis [1–6]. Amongst these, CF lung infections are often used as a model system from which one can gain insight into other respiratory infections more generally. In individuals with CF, defects in the CF trans-membrane conductance regulator gene lead to chloride retention and results in abnormally high viscosity of the mucus coating the lung epithelium. This in turn leads to impairment of the mucociliary escalator and the chronic colonisation of the mucous secretions by a number of aerobic and facultatively anaerobic bacterial species, including *Staphylococcus aureus, Haemophilus influenzae, Pseudomonas aeruginosa* and *Burkholderia cenocepacia* [1]. It is thought that rapid depletion of oxygen, resulting from metabolism by aerobic, facultatively anaerobic species and neutrophils, coupled with the highly viscous nature of the mucous secretions which impedes gas diffusion, help to create micro-environments which are conducive to the growth of a wide range of anaerobic species in the CF lung. Indeed, in recent years our understanding of bacterial infections has changed considerably, pointing to the likelihood that anaerobes may also play an important contributory role in lung infections. For example, in some CF patients with *P. aeruginosa* lung infections, anaerobes are found in numbers equal to, or exceeding those of the pseudomonads [7, 8]. Many of the anaerobes isolated from CF lungs infected with *P. aeruginosa* are normally residents of the oral cavity where they are found in sub-gingival plaque and periodontal pockets. They include members of the black-pigmenting anaerobes (BPAs) belonging to the genera *Porphyromonas* and *Prevotella*, including the species *Porphyromonas gingivalis* and *Prevotella intermedia* which have been detected by both cultural and more precise DNA-based methods, including RFLP and pyrosequencing [7–12]. The BPAs, which are major aetiological agents in the development and progression of periodontitis [13], express a large number of virulence factors [14] and have an absolute requirement for haem. Animal studies have also shown that haem up-regulates the pathogenicity of *P. gingivalis* through mechanisms including increased levels of arginine-specific gingipain proteases (Rgps) which are major virulence factors of this organism [15–17]. Growth on blood-containing media leads to cell-surface accumulation of haem in either the monomeric form (haematin) in the case of *Prevotella nigrescens* and *P. intermedia* [18] and as the μ-oxo bis haem (dimeric form) in the case of *P. gingivalis* [19]. Given the absolute haem requirements of black-pigmenting anaerobes, the fact that these species are found in high numbers in CF sputum points to a sufficiency of haem to support their growth and to enable them to avoid detection and eradication from the lung by the host. The major haem source for these species in their oral habitats (the inflamed gingival sulcus and periodontal pocket) is haemoglobin. Micro-bleeds due to inflammatory damage to the delicate epithelium result in the presence of erythrocytes in the CF lung and *P. aeruginosa* is capable of liberating haemoglobin from these cells via production of haemolysins [20,21].

The BPAs display a novel haem acquisition paradigm whereby oxyhaemoglobin is firstly oxidised to the methaemoglobin state [22–24]. In *P. gingivalis* this mechanism is mediated by the Rgps [22,23], and in *P. intermedia* by the protease interpain A (InpA) [24]. In the case of *P. gingivalis*, the more susceptible methaemoglobin substrate can be then fully proteolysed by the lysine-specific gingipain (Kgp) to release free haem [22,23]. Haem can also be extracted from methaemoglobin by the HmuY haemophore for subsequent delivery to the bacterial cell surface [25–28]. In addition, there is evidence that *P. intermedia* may help to support the growth of *P. gingivalis* by proteolytically promoting methaemoglobin formation for subsequent haem extraction by the HmuY haemophore [29]. Moreover, in the CF lung, proteases originating not only from bacteria, but also those produced by the host are considered to play an important pathophysiological role during infection. The majority of proteolytic activity originates from neutrophils [30], with neutrophil elastase the most abundant, and which has been detected in micromolar levels in bronchoalveolar lavage fluid of children with either bacterial or viral respiratory infections [31]. Recently, Cosgrove *et al*. [32] demonstrated that neutrophil as well *Pseudomonas* elastase can degrade haemoglobin, pointing to the possibility that these may be a significant factors in elevating the haem concentrations in the CF lung to levels which could support the growth of BPAs.

*P. aeruginosa* produces the blue pigment pyocyanin, a redox active phenazine compound, which displays a number of biological activities which are considered important in *P. aeruginosa* infections [33,34]. These include inhibition of the beating of cilia and hence perturbation of the mucociliary escalator [35,36]. Repeated administration of pyocyanin into the lungs of mice has been shown to cause a number of pathological changes including airway fibrosis, goblet cell hyperplasia and metaplasia, and destruction of the alveolar airspaces [37]. Pyocyanin colours blue the sputum of CF patients infected with *P. aeruginosa*, where it is found at average concentrations of 16 μg/ml (80 μM) [38]. Moreover, pyocyanin in its blue (oxidised) form when extracted from the sputum of both bronchiectasis and CF patients colonised by *P. aeruginosa* is bioavailable since it can inactivate ciliary beating *in vitro* [38]. From a clinical perspective, isolates of a highly virulent strain of *P. aeruginosa* infecting CF patients (known as the Liverpool Epidemic Strain [LES]) are typified by their over-production of pyocyanin (relative to clinical non-epidemic isolates and environmental strains), the levels of which can reach as high as 40 μM in *in vitro* cultures [39]. Importantly, Mowat *et al*. [2] have demonstrated that the presence of such *P. aeruginosa* pyocyanin over-producing strains correlates with exacerbations of CF pulmonary infection. In the leuko (reduced) form, pyocyanin can help to supply iron for *P. aeruginosa* by facilitating its release from transferrin [40,41]. However, in the oxidised form one significant biochemical property which has been observed for phenazine methosulphate is the ability to oxidise haemoglobin, forming both methaemoglobin and the higher oxidation state species ferrylhaemoglobin [42]. Given the above-mentioned haem acquisition paradigm displayed by BPAs, it follows that haemoglobin oxidation in the lung brought about by the phenazine pyocyanin, *i.e*. as a result of production by *P. aeruginosa*, may feature as an important first step in haem acquisition and in promoting virulence of co-colonising *Prevotella* and *Porphyromonas* species in the lung [7,8].

Of the BPAs, *P. gingivalis* is the best characterised with respect to its virulence properties and haem acquisition system. It is a known cause of respiratory infections [43] and animal lung infection pathogenicity models using this organism are well documented [44]. Accordingly, the possible virulence-enhancing effects of pyocyanin on *P. gingivalis* were explored with regard to its potential role in haem acquisition by employing the well-established mouse lung model [44]. Clearly whilst *P. aeruginosa* would be the source of any pyocyanin in the lung, we used in this study a mono-infection with *P. gingivalis* (plus or minus pyocyanin) to avoid any pathogenic effects arising from other redox or virulence agents produced by *P. aeruginosa* itself. In such a system, pyocyanin-mediated oxidation of oxyhaemoglobin in the lung during infectious challenge with *P. gingivalis* should lead to an increased levels of methaemoglobin and subsequently to a greater abundance of haem to support growth and up-regulate the virulence. Indeed, in this paper we show that pyocyanin can bring about methaemoglobin formation and facilitate haemoglobin degradation by neutrophil elastase and the *P. gingivalis* Kgp gingipain, as well as haem pickup by the HmuY haemophore. We also show that mice challenged with *P. gingivalis* plus pyocyanin display highly increased mortality compared to those challenged with *P. gingivalis* only. In line with our haem acquisition paradigm, we also demonstrate increased levels of both methaemoglobin and total haem in lung tissues during infection with *P. gingivalis* in the presence of pyocyanin.

## Results

### Oxyhaemoglobin oxidation by pyocyanin

**Fig 1:**
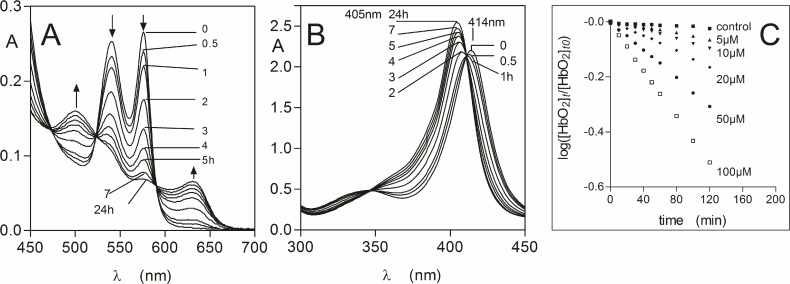
Oxidation of oxyhaemoglobin by pyocyanin. Q band (*A*) and Soret band (*B*) regions of oxyhaemoglobin (4 μM with respect to tetramer) during incubation with 20 μM pyocyanin. Buffer was 0.14 M NaCl, 0.1 M Tris-HCl, pH 7.5. Reaction was carried out at 37°C. Arrows in panel A denote changes in extinction with time related to methaemoglobin formation. The spectra were corrected for the absorbance contribution due to pyocyanin. (*C*) Rates of oxyhaemoglobin oxidation mediated by pyocyanin (5 to 100 μM). Oxidation rates were calculated as the ratio of the concentration of oxyhaemoglobin at time zero ([HbO_2_]_*t0*_) compared to that at time *t* ([HbO_2_]_*t*_), as a function of the change in A_576_ as described by Tsuruga and Shikama [35]. Control oxyHb, oxyhaemoglobin auto-oxidation.

The ability of pyocyanin to oxidise oxyhaemoglobin to methaemoglobin was tested over a concentration range which has been observed in sputa from patients infected with *P. aeruginosa* [38]. A typical set of time-dependent spectra is shown in Fig. 1A for oxyhaemoglobin incubated with 20 μM pyocyanin. These spectra displayed decreases in the absorbance of the Q bands at both 540 and 576 nm and increases at 500 and 630 nm, which are typical of the formation of methaemoglobin [45]. This was also accompanied by a blue shift in the Soret band from 414 to 405 nm (Fig. 1B). Isosbestic points were present at 410, 475, 523, and 590 nm showing that the oxyhaemoglobin had been transformed into methaemoglobin directly. When sodium dithionite (to 10 mM final concentration) was added to haemoglobin-pyocyanin incubation mixtures to concomitantly reduce the haem iron and deplete dissolved oxygen, a spectrum was generated with a 429 nm Soret and a 555 nm visible band, which is typical of deoxyhaemoglobin (data not shown). This demonstrated that methaemoglobin had originally been produced by the action of the pyocyanin on oxyhaemoglobin. Measurement of the change in A_576_ to determine the rate of oxyhaemoglobin depletion [46] revealed that the range of concentrations of pyocyanin, which might be encountered in the CF lung during *P. aeruginosa* infection, affected rapid methaemoglobin formation (Fig. 1C). The initial oxidation rates calculated by linear regression analysis over the first hour of reaction showed that even the lowest pyocyanin concentration tested (5 μM) brought about an oxidation rate 5-fold that of natural auto-oxidation, whilst this increased to 25-fold for the mid-range concentration of 50 μM pyocyanin, and was 40-fold greater for 100 μM pyocyanin (Table 1). In addition, the rate of methaemoglobin formation brought about by 50 μM pyocyanin was approximately 8-fold greater than that brought about by 50 μM mM NaNO_2_ (3.2 ± 0.07 min^-1^×10^–4^), which rapidly oxidises oxyhaemoglobin [47].

**Table 1:** Oxyhaemoglobin oxidation rates in the presence of pyocyanin.

Pyocyanin concentration (μM)	Oxidation rate (min^-1^×10^–4^)
0 (auto-oxidation)	1.1 ± 0.13
5	4.7 ± 0.11
10	7.2 ± 0.40
20	12.7 ± 0.17
50	24.8 ± 0.37
100	42.4 ± 0.29

Oxyhaemoglobin (HbO_2_) oxidation was calculated as the ratio of the concentration of oxyhaemoglobin at time zero ([HbO_2_]_*t0*_]) compared to that at time *t* ([HbO_2_]_*t*_) as a function of the change in A_576_ as described by Tsuruga and Shikama [42]. Rates were calculated from the data shown in Fig. 1 using linear regression (GraphPad Prism) over the first hour of the reaction. The initial oxyhaemoglobin concentration was 4 μM and incubations were carried out at 37°C in 0.14 M NaCl, 0.1 M Tris-HCl, pH 7.5.

### Formation of HmuY-ferrihaem complex during co-incubation of oxyhaemoglobin with HmuY in the presence of pyocyanin

**Fig 2:**
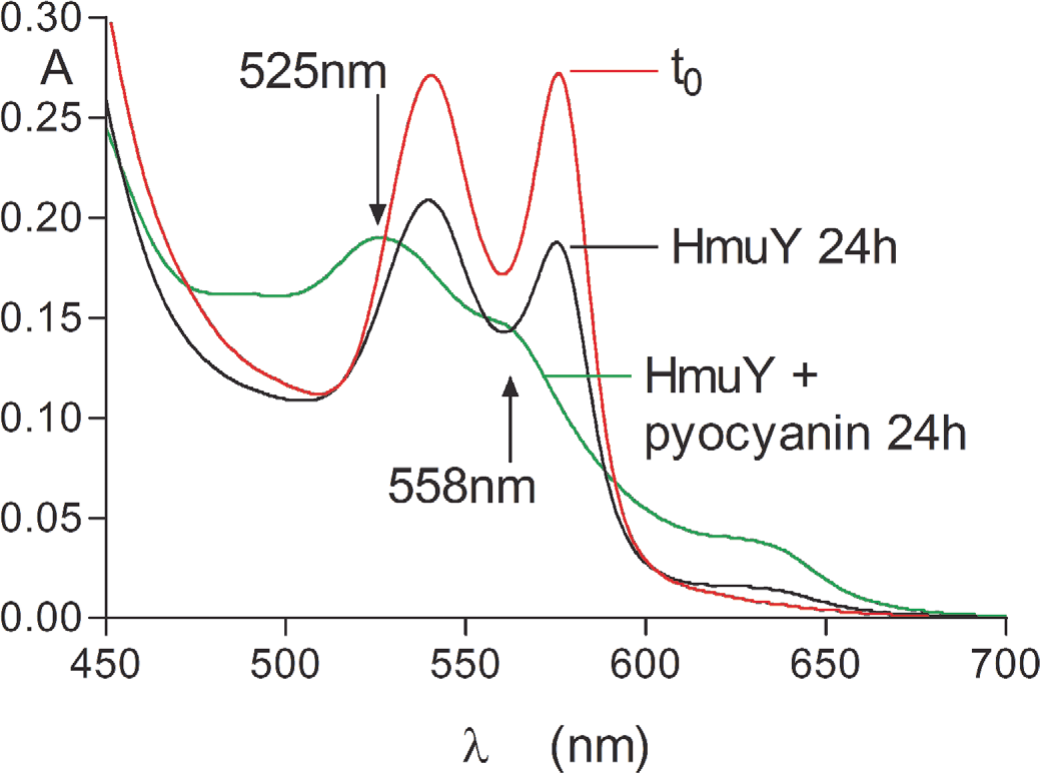
Formation of the HmuY-ferrihaem complex during co-incubation of oxyhaemoglobin with the *P. gingivalis* HmuY haemophore in the presence of pyocyanin. HmuY and pyocyanin concentrations were 32 and 50 μM, respectively, whilst that of oxyhaemoglobin was 4 μM (with respect to tetramer). Buffer was 0.14 M NaCl, 0.1 M Tris-HCl, pH 7.5. Arrows indicate the 525 nm Q band plus 558 nm shoulder related to the presence of the HmuY-ferrihaem complex, green line; oxyhaemoglobin at time zero, red line; oxyhaemoglobin plus HmuY, black line.

Given that oxyhaemoglobin oxidation is a pre-requisite step in both *P. gingivalis* gingipain-mediated haemoglobin breakdown and subsequent haem release, and the direct pickup of iron(III) protoporphyrin IX by the HmuY haemophore [22,23,28,29], it follows that agents present in the lung promoting methaemoglobin formation could facilitate both these processes and aid haem acquisition. Accordingly, the effect of co-incubating oxyhaemoglobin with HmuY in the presence of pyocyanin was examined. As seen in Fig. 2, after 24-h incubation the presence of the HmuY-ferrihaem complex was indicated by a prominent absorbance at 525 nm plus shoulder at 558 nm (green line), the positions of these bands being confirmed by producing second derivatives of the spectrum (data not shown). In comparison, these signature bands of the HmuY-ferrihaem complex were not observed in the absence of pyocyanin (black line), even after 24-h incubation. It is noteworthy that not all the haem was transferred to the HmuY after 24 h since the absorbance in the Q band region around 630 nm (Fig. 2, green line) indicated the presence of some residual methaemoglobin.

**Fig 3:**
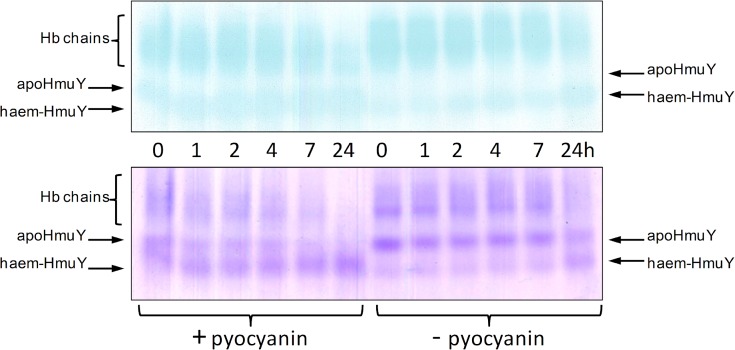
Native PAGE showing transfer of haem from oxyhaemoglobin to the HmuY haemophore during co-incubation with pyocyanin. HmuY concentration was 16 μM, whilst haemoglobin was at 4 μM (with respect to tetramer), and pyocyanin at 100 μM. Buffer was 0.14 M NaCl, 0.1 M Tris-HCl, pH 7.5, and incubation was carried out at 37°C. Upper panel, gel stained with TMB-H_2_O_2_ to reveal the presence of haem. Lower panel, gel counterstained for protein with CBB after TMB-H_2_O_2_ staining. Hb, haemoglobin; haem-HmuY, ferrihaem-HmuY complex. Each track was loaded with 4 μg of haemoglobin.

We have previously used non-denaturing PAGE followed by haem staining with TMB-H_2_O_2_ and protein staining with Coomassie Brilliant Blue (CBB) to visualise the time-dependent redistribution of haem between methaemoglobin and HmuY [28]. The pickup of haem by the haemophore is accompanied by an increase in electrophoretic mobility which is attributed to the increase in negative charge imparted to the HmuY-haem complex by the iron porphyrin carboxylates [28]. We therefore examined the effect of pyocyanin (100 μM) on haem pickup by HmuY during co-incubation with oxyhaemoglobin. As shown in Fig. 3, there was a gradual time-dependent decrease in TMB-H_2_O_2_ staining of the haemoglobin during incubation with pyocyanin. This was accompanied by an increase in haem staining associated with the HmuY showing haem transfer to the haemophore. In addition, in keeping with previous observations, there was a notable loss of protein staining of the haemoglobin chains with time [23], which is attributable to the loss of structural integrity through alpha helix unfolding [48,49] as a consequence of haem removal [50,51]. The haem staining associated with the haemoglobin chains even after 24-h incubation of haemoglobin plus HmuY and pyocyanin (Fig. 3, lower left hand panel) is also consistent with the above spectroscopical observation (Fig. 2) of the presence of some residual methaemoglobin. Note also the small degree of HmuY-haem staining in the control incubation (minus pyocyanin) after 24 h, attributable to haemophore pickup of haem from the small amount of methaemoglobin formed through natural auto-oxidation over the period of the experiment. These findings strongly corroborated the above spectroscopic data showing that pyocyanin had facilitated formation of the ferrihaem-HmuY complex by augmenting production of the methaemoglobin species.

### Neutrophil elastase breaks down methaemoglobin formed by the action of pyocyanin but not oxyhaemoglobin

**Fig 4:**
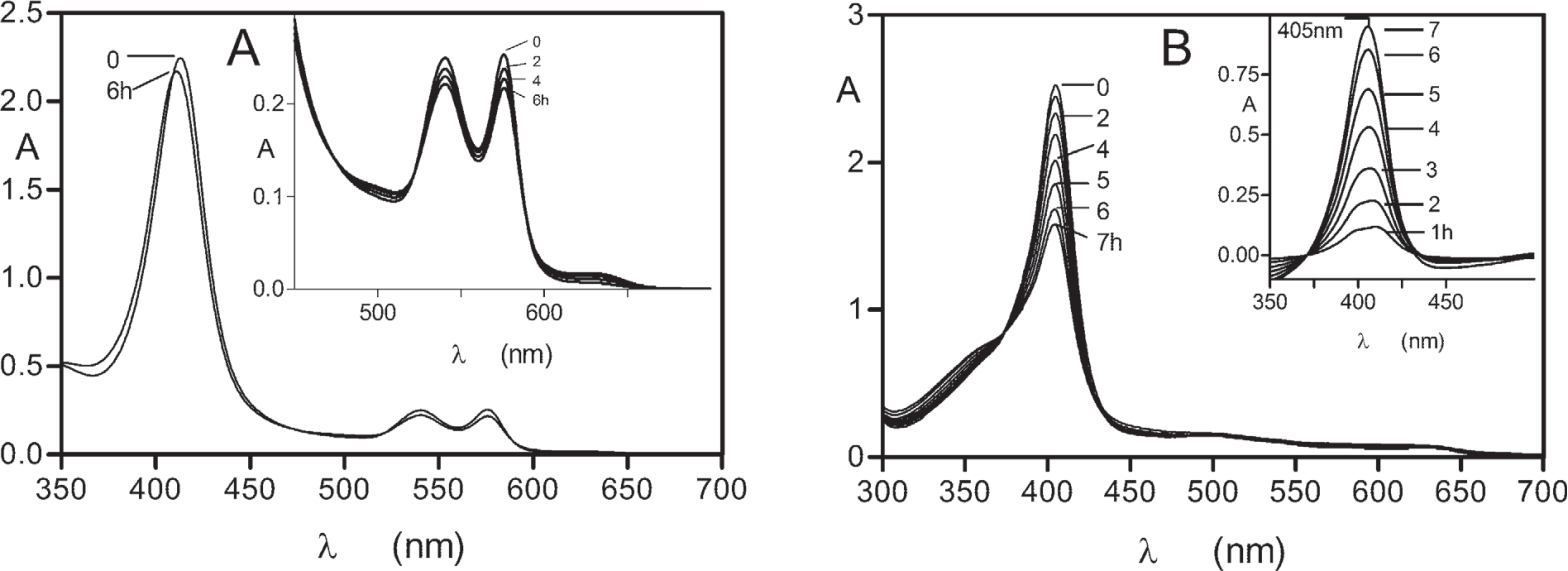
Neutrophil elastase does not breakdown oxyhaemoglobin but degrades methaemoglobin. Neutrophil elastase (2 μM) was incubated at 37°C with 4 μM oxyhaemoglobin (*A*) or with methaemoglobin (*B*), in 0.14 M NaCl, 0.1 M Tris-HCl, pH 7.5. The methaemoglobin substrate was formed by incubation of 100 μM oxyhaemoglobin with a 4-fold molar excess of sodium nitrite for 1 h at 37°C. Inset graph in panel *A*, difference spectra produced by subtraction of the elastase-methaemoglobin spectra from the control methaemoglobin spectra at each time point to show the amounts of methaemoglobin degraded with time (405 nm Soret band). Inset graph in panel *B*, difference spectra made by subtracting the enzyme incubation spectra from those of the control methaemoglobin spectra at each time period to show the incremental amounts of methaemoglobin degraded.

A ubiquitous feature of lung infections, including those of CF patients, is the presence of neutrophil-derived proteases as a consequence of chronic and acute inflammation. Of these, neutrophil elastase has been shown to be the most abundant, reaching levels as high as 2 μM in the bronchoalveolar lavage fluids from CF patients with lung infections [31,32]. We therefore firstly examined the effect of neutrophil elastase on oxyhaemoglobin. Although the protease (at 2 μM) was able to mediate a small degree of haemoglobin oxidation during a 6-h incubation period (Soret band blue shift to 406 nm, plus decreases in A_576_ and A_541_, and increases in A_500_ and A_630_) (Fig. 4A), loss of Soret band intensity, which would be indicative of protein breakdown and haem release, was not seen. Using the decrease in A_577_ to determine the relative level of methaemoglobin formation, it was found after 6 h, that the rate of oxidation was lower than the control auto-oxidation (data not shown). Cosgrove *et al*. [32] recently found that neutrophil elastase and *Pseudomonas* elastase could break down haemoglobin. However, this is likely explained by the nature of the commercial substrate they used, which is known to contain a high proportion of the protein in the methaemoglobin form. To confirm this, we examined the ability of neutrophil elastase to break down methaemoglobin induced by treatment with sodium nitrite. As shown by the rapid decrease in Soret band intensity (Fig. 4B), 2 μM neutrophil elastase was effective in breaking down nitrite-induced methaemoglobin and releasing haem. This effect was also seen down to a protease concentration of 0.25 μM (data not shown). Under these conditions, the spectrum of the product formed by the action of the neutrophil elastase after 24 h had a 393 nm Soret band plus shoulder at 360 nm (data not shown), which is typical of free iron(III) protoporphyrin IX in the μ-oxo dimeric form [52,53]. The series of difference spectra produced by subtracting each neutrophil elastase-methaemoglobin spectrum from the control methaemoglobin spectrum at each incubation time period (Fig. 4B, inset graph) showed that the enzyme had progressively degraded the methaemoglobin substrate (405 nm Soret bands). These data contrast starkly with the inability of neutrophil elastase to bring about oxyhaemoglobin breakdown when the haem iron was in the Fe(II) oxidation state, even at the highest protease concentration examined.

### Neutrophil elastase degradation of methaemoglobin formed by the action of pyocyanin

**Fig 5:**
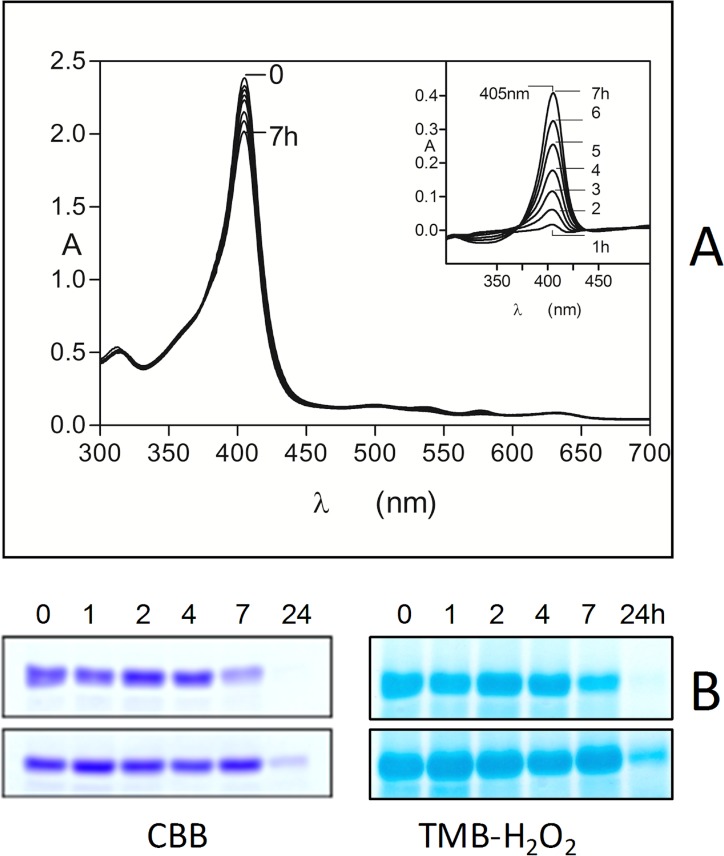
Spectroscopic (A) and SDS-PAGE (B) demonstration of the breakdown of pyocyanin-induced methaemoglobin by neutrophil elastase. Methaemoglobin was formed from oxyhaemoglobin (100 μM with respect to tetramer) by incubation with 50 μM pyocyanin for 7 h. Total haemoglobin in the assay was 4 μM and neutrophil elastase was at 2 μM, and digestions were carried out at 37°C in 0.14 M NaCl, 0.1 M Tris-HCl, pH 7.5 at 37°C. SDS-PAGE was carried out under non-reducing conditions and gel tracks are loaded with 4 μg haemoglobin. Inset in panel A, difference spectra produced by subtracting the neutrophil elastase-methaemoglobin spectrum from the control methaemoglobin spectrum at each incubation time point to show the progressive breakdown of the methaemoglobin substrate. Panel B, pyocyanin-induced methaemoglobin plus neutrophil elastase (uppermost gels) and lower gels, control auto-oxidised methaemoglobin plus neutrophil elastase. Gels were firstly stained with TMB-H_2_O_2_ to reveal the presence of haem and then counterstained for protein with CBB.

Given the susceptibility of nitrite-induced methaemoglobin, it follows that pyocyanin-mediated oxidation would also yield methaemoglobin susceptible to neutrophil elastase. Predictably, we found that the pyocyanin-induced methaemoglobin was also degraded as shown by the decrease in the Soret band absorbance (Fig. 5A). This was confirmed by examining the difference spectra as above for the NaNO_2_-induced methaemoglobin substrate (Fig. 5A, inset graph). However, the amount of methaemoglobin degraded was lower than that of the nitrite-induced methaemoglobin. Moreover, after 7-h incubation, addition of 10 mM sodium dithionite yielded a deoxyhaemoglobin spectrum, with no evidence of a haemoglobin haemochrome present (data not shown). To test if the lower susceptibility of pyocyanin-induced methaemoglobin was due to the presence of pyocyanin, we determined whether it could inhibit the elastase-catalysed hydrolysis of Azocoll and the synthetic substrate MeOSuc-Ala-Ala-Pro-Val-pNA. We found that at a physiologically relevant concentration (100 μM) [38] pyocyanin had no significant effect on neutrophil elastase activity (data not presented). However, we confirmed the susceptibility of the pyocyanin-induced methaemoglobin substrate to neutrophil elastase using non-reducing SDS-PAGE (Fig. 5B). Despite the apparent lower susceptibility of methaemoglobin formed by pyocyanin towards neutrophil elastase compared to that induced by nitrite (as shown by spectroscopy), we found that the protease brought about progressive reductions in CBB and TMB-H_2_O_2_ staining of the protein over a 24-h period (Fig. 5B, upper gels), indicating both protein breakdown and haem loss. In comparison, little neutrophil elastase degradative activity was seen against a sample of control haemoglobin auto-oxidised for 7 h (Fig. 5B, lower gels).

### Synergistic activity of pyocyanin and K-gingipain in mediating haem release from oxyhaemoglobin and in HmuY-haem complex formation

**Fig 6:**
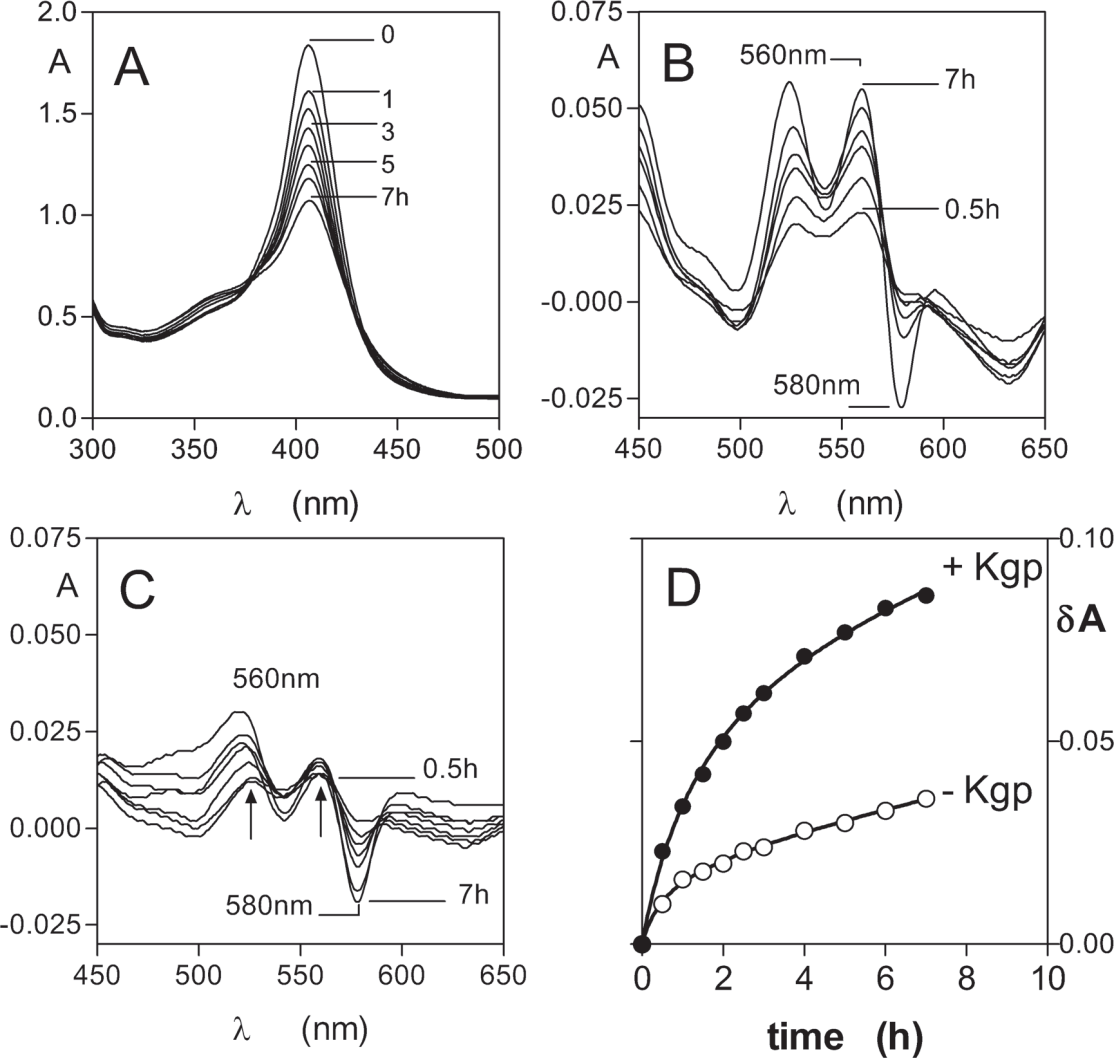
Kgp degrades pyocyanin-induced methaemoglobin and facilitates haem transfer to HmuY. (*A*) Pyocyanin-induced methaemoglobin (4 μM with respect to tetramer) was incubated with 1 μM Kgp at 37°C in 0.14 M NaCl, 0.1 M Tis-HCl, pH 7.5, and the methaemoglobin Soret band intensity monitored with time. Visible spectral regions showing the formation of the HmuY-haem complex after incubation of pyocyanin-induced methaemoglobin with HmuY in the presence (*B*) and absence (*C*) of Kgp are shown. HmuY concentration was 16 μM, and incubation conditions as in (*A*). (*D*) Change with time of the absorbance between the Q band at 560 nm and the trough at 580 nm as a measure of the formation of HmuY-haem complex from pyocyanin-induced methaemoglobin in the presence and absence of Kgp.

Given the role of Kgp in haemoglobin breakdown and formation of the μ-oxo bishaem component of the pigment [22,23], and the fact that Kgp enhances the haem transfer from methaemoglobin to HmuY [28], it was appropriate to examine its ability to breakdown methaemoglobin formed via the action of pyocyanin. We observed that Kgp mediated a rapid breakdown of methaemoglobin as evidenced by the loss of Soret band absorbance (Fig. 6A). Since Kgp degraded pyocyanin-induced methaemoglobin, it follows that a similar synergistic effect should operate for HmuY haem binding from this substrate. Pyocyanin-induced methaemoglobin (4 μM with respect to tetramer) was incubated with 16 μM HmuY in the presence and absence of 1 μM Kgp. For both incubations we observed Soret band red shifts to 411 nm and changes in the visible bands with time, indicative of formation of the HmuY-haem complex. These visible band absorbance changes were more apparent when difference spectra were made by subtracting the time-zero sample from the subsequent spectra at each time point which revealed spectra with bands at 528 and 558 nm (Fig. 6B), which gradually grew in intensity. In contrast, the degree of HmuY-haem complex from pyocyanin-induced methaemoglobin was minimal in the absence of Kgp (Fig. 6C). To quantify the degree of HmuY-haem complex formation, the absorbance change between the peak at 560 nm and the trough at 580 nm was plotted against time (Fig. 6D). This showed a larger amount of HmuY-haem complex formation from pyocyanin-induced methaemoglobin in the presence of Kgp.

In contrast to neutrophil elastase, *Pseudomonas* elastase was relatively inefficient in degrading methaemoglobin formed by the action of pyocyanin as seen by the low degree of Soret band loss. From this we calculated that the *Pseudomonas* elastase degraded only 54% of the methaemoglobin compared to that mediated by neutrophil elastase over the same time period (7 h) under the same conditions as described in Fig. 5 (data not shown). It is noteworthy that Smith *et al*. [54] found that *Pseudomonas* elastase expression was down-regulated in strains derived from adult patients who were chronically infected with *P. aeruginosa*. Coupled with the above observations on *Pseudomonas* elastase, it is likely that early in establishment of infection, *P. gingivalis* may be reliant upon neutrophil elastase to engender an initial supply of free haem by degrading methaemoglobin before sufficient gingipain expression can take on the role of aiding haem acquisition through HmuY haemophore deployment.

### Mouse lung infection model

**Fig 7:**
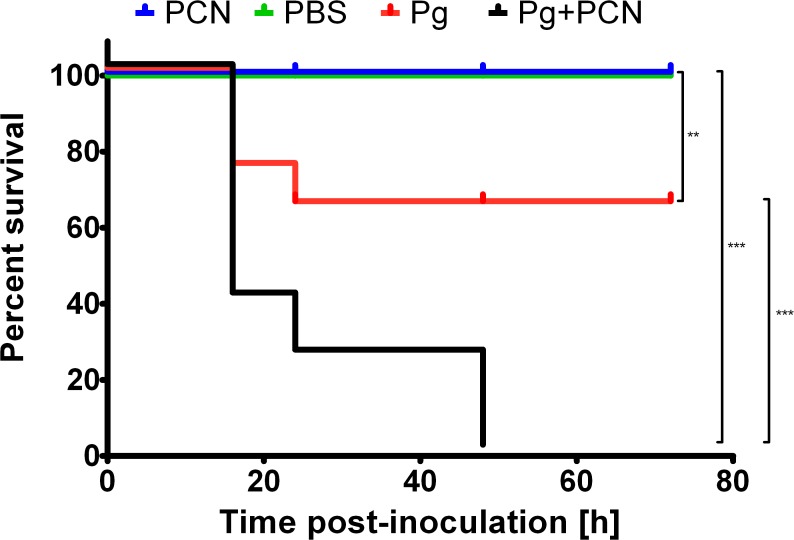
Kaplan-Meier plots showing differences in survival of C57/BL/6 mice after intra-tracheal challenge with *P. gingivalis, P. gingivalis* plus pyocyanin, PBS, and PBS plus pyocyanin. Mice (10 animals per group) were inoculated with 3×10^9^ CFU of wild-type *P. gingivalis* W83 (Pg) or *P. gingivalis* plus 40 μg pyocyanin (Pg+PCN) into the lungs *via* the trachea. Control groups were challenged with PBS or with PBS plus 40 μg pyocyanin (PCN). The survival of mice was monitored during whole time of procedure. Log-rank test, n = 10 in all cases, **P<0.01 and ***P<0.001 for control PBS or PBS+PCN-inoculated, and *P. gingivalis*- or *P. gingivalis*+PCN-inoculated animals, respectively.

In these experiments we evaluated the effect of pyocyanin, either alone or in combination with *P. gingivalis*, in the development of pulmonary infection in C57/BL/6 mice in line with the mono-infection model described by Nemec *et al*. [44]. Importantly, this avoided any complications which would arise from the pathogenic effects of using a co-infection with *P. aeruginosa* as a source of pyocyanin. Clinical symptoms of pneumonia and myeloperoxidase activity, as a measure of neutrophil activation in the lung tissue, were assessed. The course of *P. gingivalis* W83 infection with or without pyocyanin, in terms of mouse survival is illustrated in Fig. 7. Mice intra-tracheally inoculated with PBS or with PBS plus pyocyanin remained healthy throughout the observation period. However, 30% of mice inoculated with *P. gingivalis* alone, and 70% of mice inoculated with *P. gingivalis* plus pyocyanin died within 20 h after infection. These differences were statistically significant and were even more profound at longer times post-infection. While all animals infected with *P. gingivalis* in the presence of pyocyanin were dead by 48 h, no more casualties were observed in the group inoculated with *P. gingivalis* alone up to the end of the experiment (72 h).

**Fig 8:**
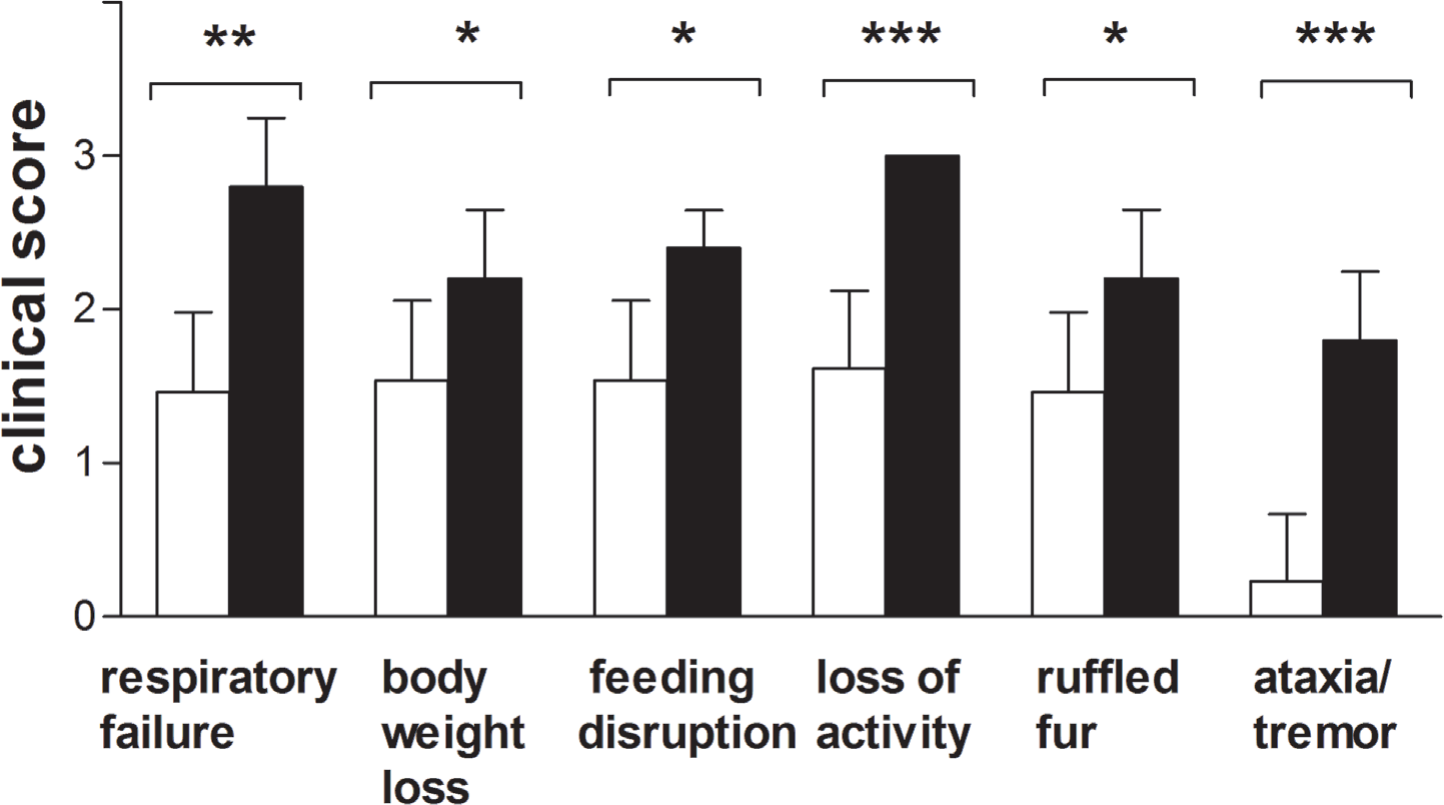
Clinical evaluation scores of mice (on a scale of 0 to 3) after intra-tracheal inoculation with 3×10^9^ CFU *P. gingivalis* W83, or 3×10^9^ CFU *P. gingivalis* W83 + 40 μg pyocyanin. Filled bars, mice challenged with *P. gingivalis* plus pyocyanin. Statistical significance: *P<0.01; **P<0.05; ***P<0.005.

Mice challenged either with *P. gingivalis*, or *P. gingivalis* plus pyocyanin demonstrated evidence of clinical infection characterized by respiratory failure, weight loss, ruffled fur, disruption to feeding, loss of activity, and ataxia/tremor (Fig. 8), signs that are associated with an ongoing infection. However, symptoms of pneumonia were significantly less developed in mice inoculated with *P. gingivalis* only. This was most clearly seen in the case of loss of activity and ataxia/tremor. The last symptom was barely observed in mice infected with *P. gingivalis* alone. Cumulatively these results clearly indicated that pyocyanin enhances virulence of *P. gingivalis* in the infected lungs.

### Assessment of viable organisms in the lungs of infected mice

**Fig 9:**
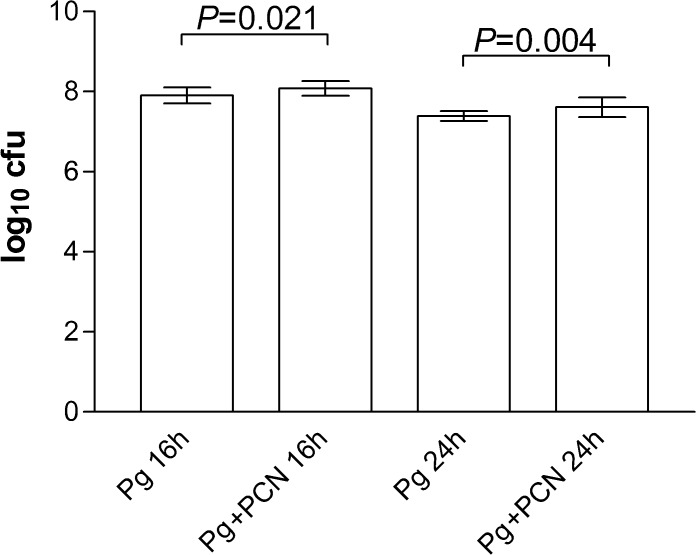
Viable cell counts (CFU) of *P. gingivalis* W83 in the lungs of mice challenged with bacteria in the presence and absence of pyocyanin. Mouse lungs were homogenized in PBS and 10-fold serial dilutions plated onto horse blood agar and incubated anaerobically. NS, not statistically significant. Pg, *P. gingivalis* alone; Pg+PCN, *P. gingivalis* plus 40 μg pyocyanin.

The enhanced morbidity and mortality induced by *P. gingivalis* plus pyocyanin can be due to increased survival and/or proliferation of bacteria in the lungs. Therefore homogenates of lung collected at 16 h and 24 h post inoculation were plated and the CFU of *P. gingivalis* counted after anaerobic culture. Surprisingly there was no significant difference in the CFU count between the groups (Fig. 9). Collectively, these data show that the presence of pyocyanin when administered along with *P. gingivalis* did not affect colonization, survival and proliferation of bacteria in the lungs. Instead our results suggest that the enhanced pathogenicity of *P. gingivalis* in the presence of pyocyanin is due to increased secretion of virulence factor(s) rather than the improved fitness of bacteria *per se* in the infected lungs.

### Myeloperoxidase activity as a measure of neutrophil activation

**Fig 10:**
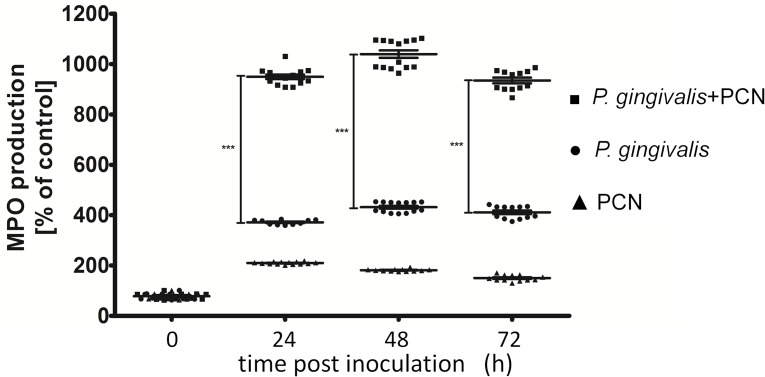
Myeloperoxidase activities (MPO) in homogenates of mouse lungs challenged with *P. gingivalis, P. gingivalis* plus pyocyanin, or pyocyanin alone. The MPO activity was expressed as % of control activity (mice inoculated with PBS only). The data points represent the mean ± SD from two independent MPO assays performed for each group (PBS+PCN, *P. gingivalis*, and *P. gingivalis*+PCN). The number of animals in the PBS and PBS+PCN groups was 10, while those in the *P. gingivalis* and *P. gingivalis*+PCN groups was 8 and 4 (at 24 h), 7 and 3 (at 48 h), and 7 and 0 (at 72 h), respectively. Statistical significance: ***P<0.005.

One of the well characterized effects of gingipain proteases on the host is enhancement of neutrophil attraction by increasing chemotactic activity of IL-8 [55–57] and neutrophil activation via the PAR-2 pathway [58]. Excessive amounts of activated neutrophils in the tissues may have a deleterious effect on lung biology and explain the difference in mortality/morbidity between mice infected with *P. gingivalis* and those challenged with *P. gingivalis* in the presence of pyocyanin. To assess this we measured myeloperoxidase activity which is a marker of neutrophil activation and accumulation in tissues during inflammation as a result of infection [59]. This activity was assayed in the lung tissues from surviving mice in each experimental group of animals at 24, 48 and 72 h after inoculation. Protein extracts of the lung homogenates showed significantly higher myeloperoxidase levels in animals inoculated with *P. gingivalis* or with *P. gingivalis* plus pyocyanin, compared with mice inoculated with PBS or PBS plus pyocyanin only (Fig. 10). Importantly, however, compared with infection with *P. gingivalis*, inoculation with *P. gingivalis* plus pyocyanin was associated with the much higher myeloperoxidase activities and hence the greatest neutrophil influx into the lung. These myeloperoxidase levels were measured only at 24 h since at later times post-challenge no mice were alive which had been infected with *P. gingivalis* plus pyocyanin (Fig. 7). It is important to note here that neutrophil influx as a measure of inflammation correlated with significantly increased clinical evaluation scores and hence severity of infection.

### Levels of methaemoglobin and total haem in mouse lung homogenates

**Fig 11:**
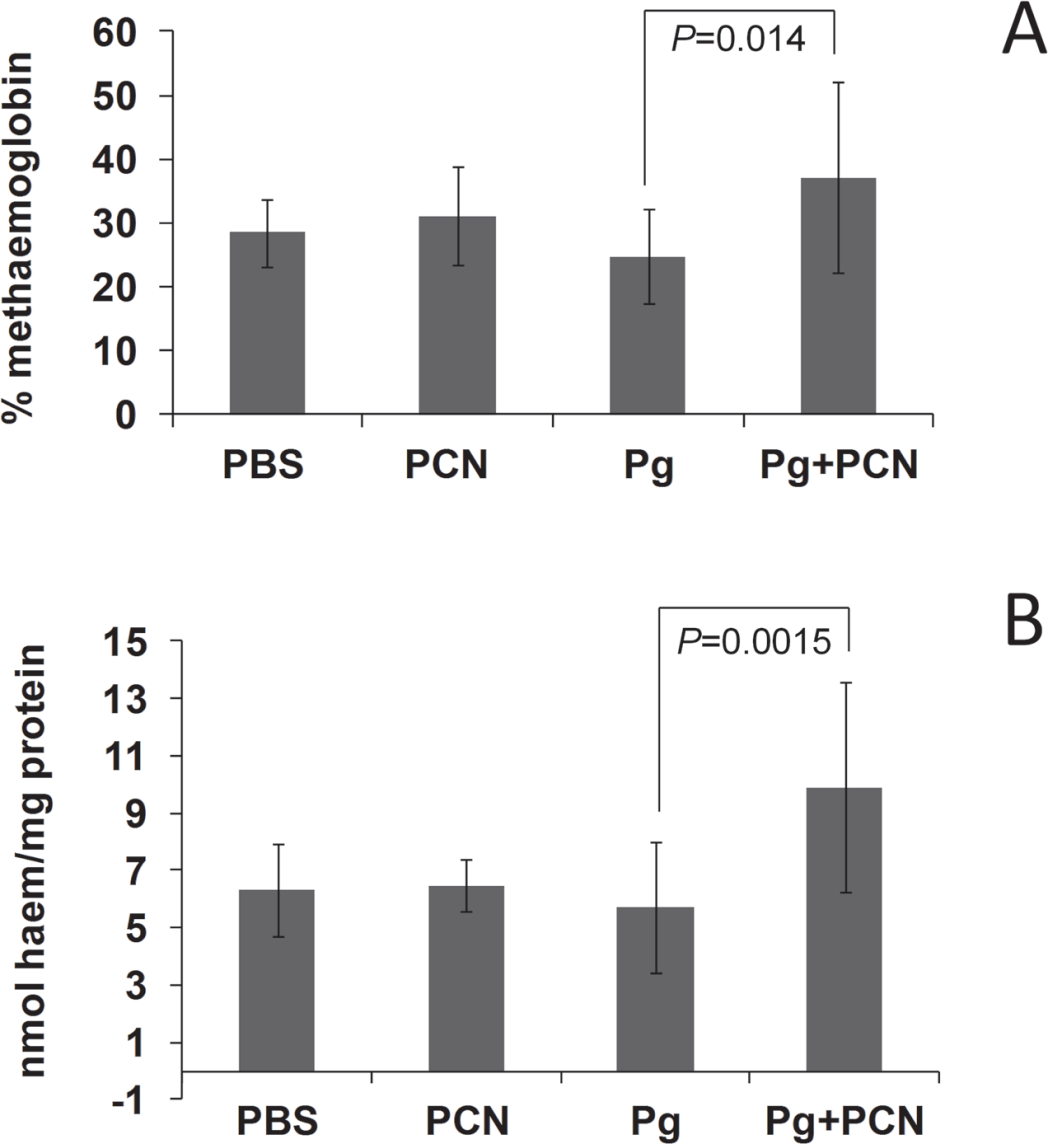
Methaemoglobin and total haem levels in homogenates of mouse lung tissues 24 h after intra-tracheal challenge with *P. gingivalis, P. gingivalis* plus pyocyanin, or pyocyanin alone. (*A*) Percentage methaemoglobin compared to that of oxyhaemoglobin in the soluble protein fraction of the lung homogenates. (*B*) Total haem levels in the homogenate fraction comprising the insoluble tissues and bacteria. Lungs were homogenised, diluted with PBS, clarified by centrifugation, and the UV-visible spectra of the supernatant fractions recorded immediately to determine the oxyhaemoglobin and methaemoglobin concentrations by multicomponent analysis from A_576_ and A_630_ values as previously described [12]. The methaemoglobin levels are expressed as a percentage of the total haemoglobin present (oxyhaemoglobin plus methaemoglobin). The pellets after the centrifugation step containing insoluble tissue and bacteria were solubilised in 0.1 M NaOH in a bath sonifier and assayed for total haem and protein. The haem data are expressed as nmol haem per mg protein. Experimental groups challenged with phosphate-buffered saline (PBS), pyocyanin (PCN), *P. gingivalis* only (Pg), and *P. gingivalis* plus pyocyanin (Pg+PCN). Kruskal-Wallis one-way analysis of variance, statistical significance: **P < 0.01; NS, not statistically significant.

Concentrations of both oxyhaemoglobin and methaemoglobin were measured spectrophotometrically in the lung homogenates which had been clarified by centrifugation. This enabled calculation of the relative percentages of oxy- and methaemoglobin species present. We found that the mean percentage of methaemoglobin in lung homogenates from control PBS-inoculated animals was 28.5 ± 5.3 (n = 11), but this was not statistically different from mice inoculated with PBS plus pyocyanin (31.1 ± 7.8; n = 13). However, there was a significant elevation (*P* = 0.014) in the percentage of methaemoglobin present in lung homogenates from mice challenged with *P. gingivalis* plus pyocyanin (37.1 ± 14.8; n = 15) compared to those challenged with *P. gingivalis* alone (24.7 ± 7.4; n = 19) (Fig. 11A).

In addition, we assayed for total haem in the supernatant fractions from the lung homogenates and found that these correlated with the total haemoglobin concentrations, *i.e*. both methaemoglobin and oxyhaemoglobin combined (data not shown). As discussed above, methaemoglobin represents the primary substrate from which haem is acquired by *P. gingivalis via* the actions of Kgp and HmuY [22,23]. Given that pyocyanin, when administered along with *P. gingivalis* resulted in an elevation of the level of methaemoglobin in the soluble fraction from the lung homogenates (Fig. 11A), it may be anticipated that this would lead to an increase in haem acquisition by the bacterial cells which would be found pelleted along with the insoluble lung tissues (after centrifugation at 18,000×*g* for 20 min) rather than in the supernatant fraction. Therefore the total haem present in the homogenate pellets relative to the total protein was measured (Fig. 11B). We observed statistically significant differences between mice challenged with *P. gingivalis* plus pyocyanin and those challenged with *P. gingivalis* only (*P* = 0.0015). Thus, although pyocyanin instilled into mouse lungs alone did not itself increase the level of methaemoglobin, there was a significant increase in the amount of methaemoglobin as a percentage of the total haemoglobin in the lung homogenates when pyocyanin was administered alongside *P. gingivalis*. This is not surprising since the bacterial infection alone, which demonstrably lead to inflammation (as manifested by elevated levels of neutrophil myeloperoxidase), would lead to micro-bleeds and hence free haemoglobin. This, in turn, would be rapidly oxidised in the presence of pyocyanin and, in the methaemoglobin form, would be more easily utilised, *i.e. via* direct extraction of the ferrihaem by the HmuY haemophore and degraded by neutrophil elastase or by *P. gingivalis* Kgp as demonstrated above. We would thus conclude that although *P. gingivalis* itself is capable of causing infection, the presence of pyocyanin considerably augments haem availability by promoting formation of methaemoglobin from which haem can be released by the action of the Kgp gingipain.

### Gingipain activity in mouse lung homogenates

**Fig 12:**
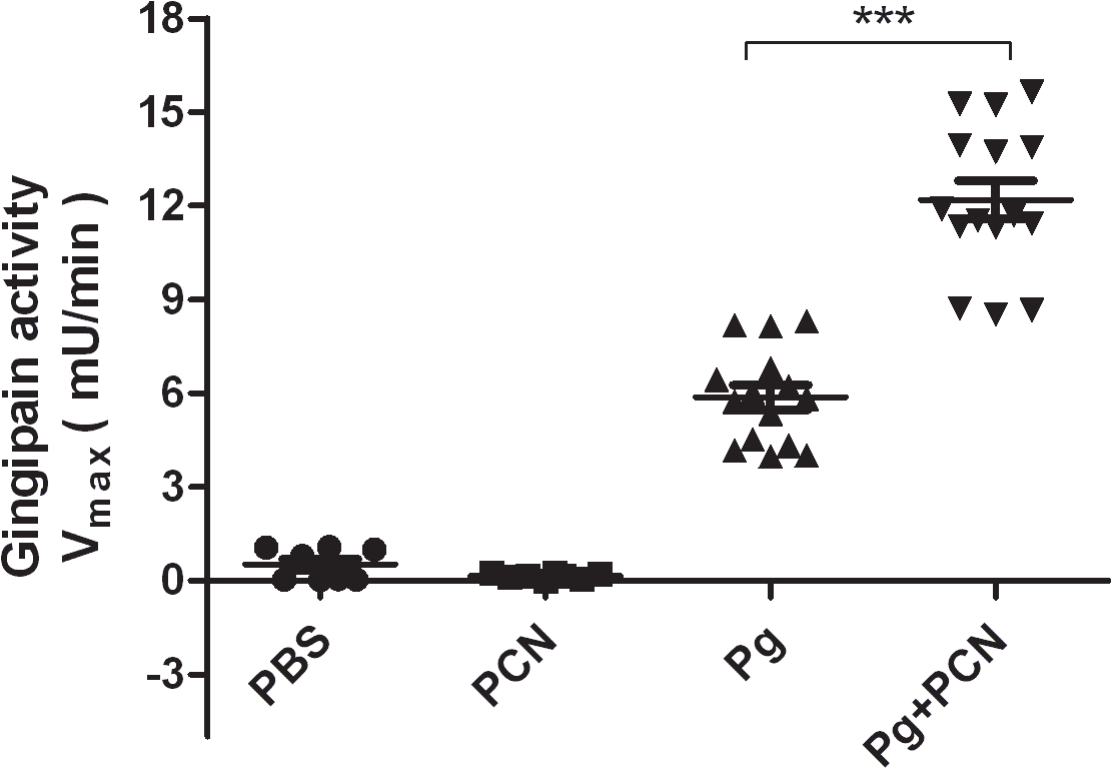
Rgp activity in the homogenates of lung tissue. Total Rgp activity was measured using BApNA as substrate. See text for details. Student's *t*-test with Welch’s correction, statistical significance: *** *P*<0.0001.

Gingipains are the most important virulence factors directly or indirectly (*e.g.*, involved in fimbriae assembly) in all aspects of *P. gingivalis* virulence. It is well documented that *P. gingivalis* grown in haem-excess conditions up-regulates gingipain activity [16,60,61], and in the context of increased levels of methaemoglobin and haem in mouse lungs challenged with *P. gingivalis* plus pyocyanin, we postulated that elevated haem concentrations during infection may influence gingipain protease expression. To experimentally verify this possibility we measured total Rgp activities in the homogenates of mouse lungs. Gingipain activity was at low baseline levels in control mice receiving either pyocyanin or PBS (Fig. 12). Remarkably, however, and compliant with our hypothesis, significantly higher R-gingipain activity was detected in the lungs of animals which had been challenged with *P. gingivalis* plus pyocyanin, compared to those inoculated with *P. gingivalis* only (Fig. 12).

### Effect of pyocyanin on growth and gingipain protease activity of *P. gingivalis in vitro*

**Fig 13:**
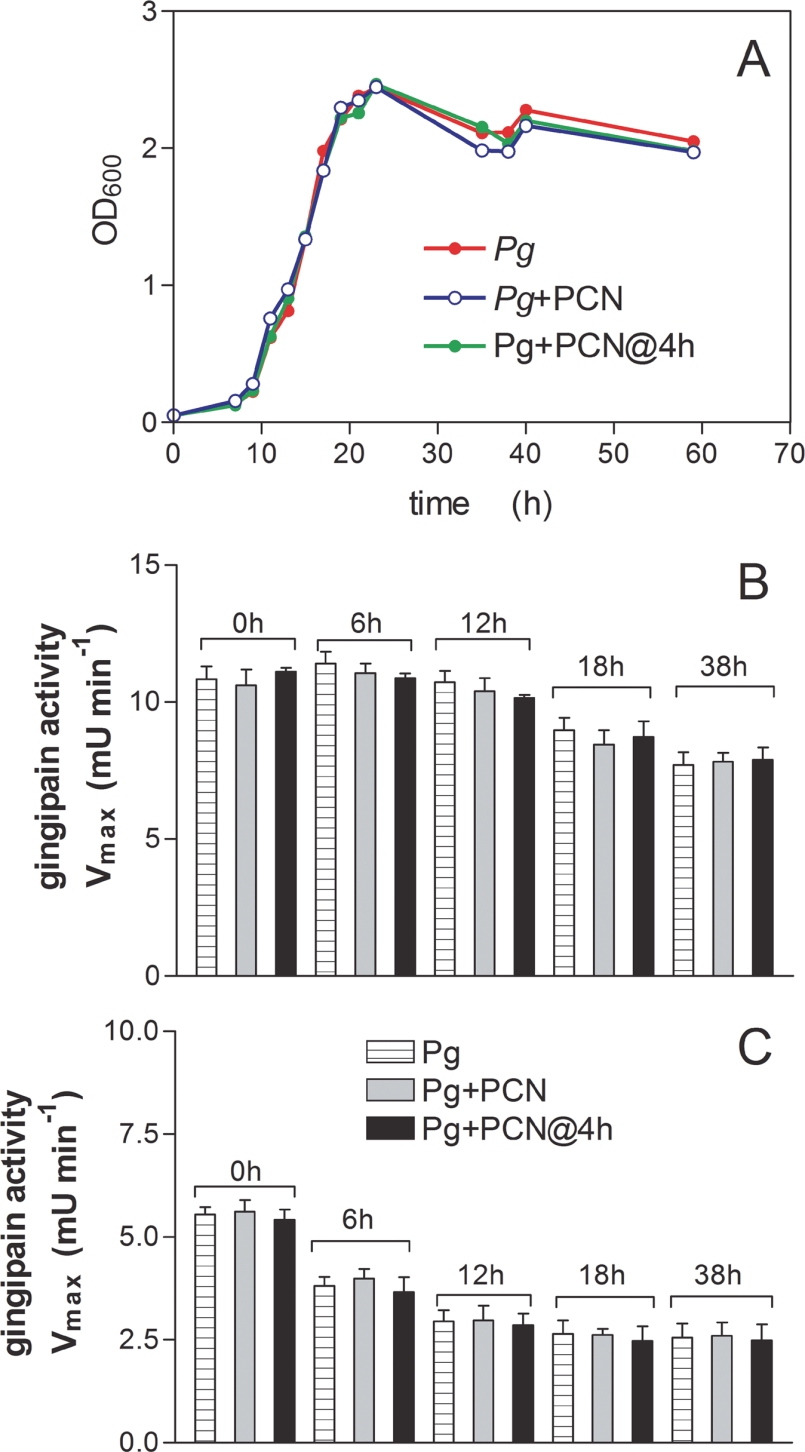
Effect of pyocyanin on the growth (A) and on cell-associated (B) and extracellular (C) R-gingipain protease activity of *P. gingivalis* in batch culture. (*A*) *P. gingivalis* strain W83 was grown in the absence and presence of pyocyanin which was added at the start or in the lag phase of growth. Growth was measured by OD_600_ on duplicate samples and the average of the two is shown. (*B* and *C*) *P. gingivalis* W83 was grown in the absence and presence of pyocyanin as in (*A*) and the cultures were sampled at various times and assayed for both cell-associated (*B*) and extracellular (**C**) R-gingipain activity using the substrate BApNA after the bacteria were pelleted by centrifugation. The protease assays on bacterial cells and extracellular supernatant fractions at each time period were performed on six replicate samples and data are shown as mean ± SD. *Pg*+PCN, *P. gingivalis*+pyocyanin at start of incubation; *Pg*+PCN@4 h, *P. gingivalis* + pyocyanin added 4 h after start of incubation; *Pg, P. gingivalis* minus pyocyanin.

It was also observed that pyocyanin did not affect the growth of *P. gingivalis in vitro* when added to the bacterial cultures either at the beginning of incubation or during the lag phase of growth (Fig. 13A). In addition, pyocyanin had no effect on cell-associated or extracellular R-gingipain activity from the bacterial cultures at the early-, middle-, late-logarithmic, or stationary phases of growth (Fig. 13B and 13C).

### Growth of *P. gingivalis* in the vicinity of *P. aeruginosa* on solid media

**Fig 14:**
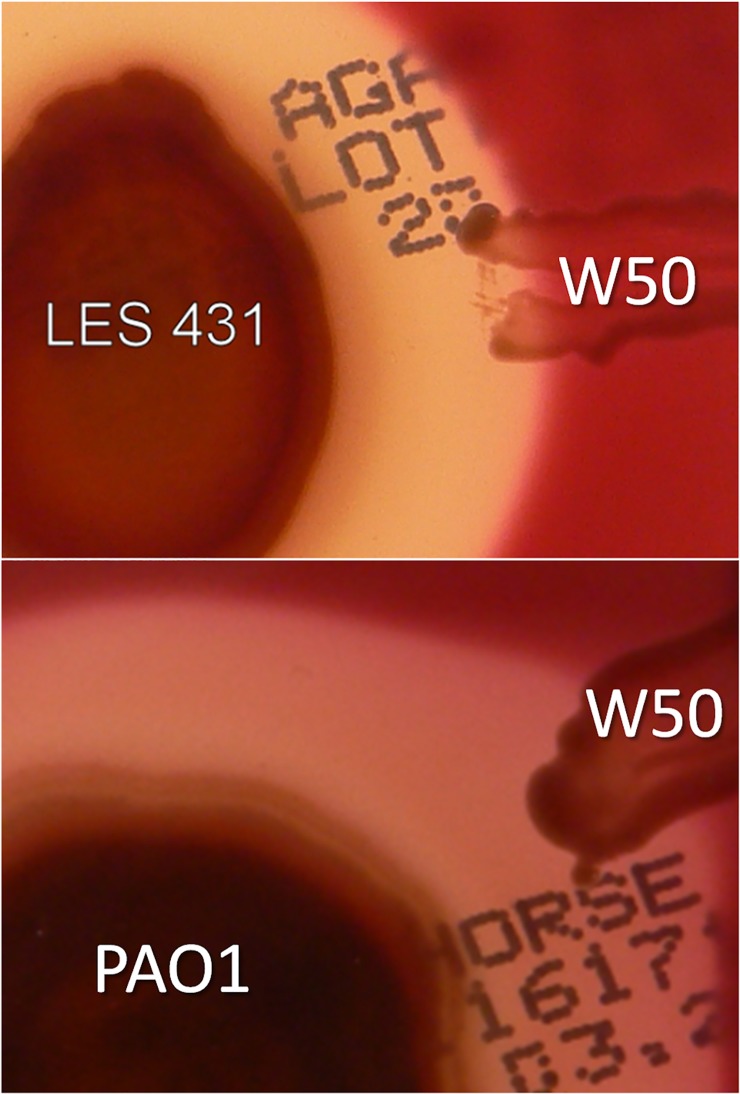
Growth of *P. gingivalis* on blood agar in the presence of secreted products of *P. aeruginosa*. Columbia horse blood agar was inoculated with pyocyanin producing *P. aeruginosa* strains PAO1 and LES 431, and after overnight growth at 37°C, *P. gingivalis* was was streaked radially close to but not touching the *P. aeruginosa* growth, and incubated for 4 days anaerobically.

We tested the ability of *P. gingivalis* to grow in the presence of the secreted products of *P. aeruginosa* on blood agar. Pyocyanin producing strains PAO1 and LES 431 were grown as a single large colony overnight at 37°C by which time blue colouration was seen as halos around each strain. *P. gingivalis* was then streaked radially a few mm from, but not touching, each *P. aeruginosa* colony, and the plates were incubated for 4 days anaerobically. We observed that *P. gingivalis* grew and pigmented normally in the vicinity of both strains (Fig. 14). This indicated that *P. gingivalis* was capable of growing in the presence of extracellular products secreted by *P. aeruginosa*.

## Discussion

From a physiological perspective it is thought that the highly viscous nature of sputum, which limits gaseous diffusion, plus the metabolic activities of aerobic and facultative anaerobic species and neutrophils, which further deplete the oxygen levels, create micro-environments which are conducive to the growth of anaerobic bacteria in the infected lung, mostly in patients with CF [62,63]. However, the fact that oral BPAs with their obligate requirement for haem are found in the infected lung *de facto* indicates that sufficient haem must be available to support their growth. The richest sources of haem in the infected lung are erythrocytes released from micro-bleeds due to inflammatory damage to the delicate epithelium. Importantly, *P. aeruginosa* produces potent haemolysins capable of releasing haemoglobin from erythrocytes [20,21], and proteomic studies have identified haemoglobin-derived peptide fragments in CF sputum indicating that haemoglobin is indeed actively degraded in this environment [64]. Whilst haemoglobin levels have not been quantified in the CF lung, micro-bleeds into the airways could locally elevate haem levels to around 8 mM (*i.e.*, the concentration of haem present as haemoglobin in whole blood should all the haem be liberated from the protein). This represents a sufficiency of haem which could enhance the virulence of *P. gingivalis* [15]. In addition, in keeping with their obligate haem requirements, both *P. gingivalis* and *P. intermedia* are also able to release haemoglobin from erythrocytes *via* expression of haemolysins and other proteases [65–68].

For BPAs the key to utilisation of haemoglobin as a haem source lies in their ability to mediate haemoglobin haem-iron oxidation [17–19]. Normally, upon release from haemolysed erythrocytes the haem iron of oxyhaemoglobin undergoes a small degree of auto-oxidation to the methaemoglobin form (HbFe^+^O_2_). This is a result of loss of intra-erythrocyte glutathione and methaemoglobin reductases, which reinstate its functionality as an oxygen carrier by re-reducing the haem iron to the Fe(II) state, and through the effect of dilution which results in haemoglobin dissociation into αβ dimers, which display increased oxidation compared to the intact tetrameric protein [69]. However, in neutral to alkaline pH environments (as may be encountered in the diseased periodontal pocket and the lung) the natural auto-oxidation rates of haems in the oxyhaemoglobin tetramer and αβ dimers are at their lowest [70]. Thus, to satisfy haem requirements and to reach high numbers in the CF lung [7,8], BPAs must be reliant upon a mechanism, which would ensure effective haemoglobin oxidation. The production of pyocyanin by *P. aeruginosa* appears to be a phenotype common to CF isolates [71]. Relative to clinical non-epidemic and environmental strains, cultures of pyocyanin over-producing clinical isolates (such as *P. aeruginosa* LES), have been shown to generate pyocyanin levels as high as 40 μM *in vitro* [39]. Pyocyanin is abundant in the sputa of CF and bronchiectasis patients whose lungs are infected with *P. aeruginosa*, where average concentrations of around 80 μM have been observed [38]. The central role played by pyocyanin in pathogenicity is demonstrated by the fact that pyocyanin-deficient mutant strains of *P. aeruginosa* display attenuated virulence in acute and chronic mouse lung infection models [33]. Besides perturbing host cellular functions [34], including damage to lung epithelial cells [72], pyocyanin and other phenazine compounds also act as antibiotics [73]; these effects being linked to the generation of superoxide and hydrogen peroxide [74,75]. However, *P. gingivalis* is resistant to reactive oxygen species-dependent antimicrobial activity of the host [76]. In keeping with this, we found that growth and gingipain production by *P. gingivalis* in liquid culture was unaffected by the presence of pyocyanin (Fig. 13). Moreover, we observed that it can also grow and pigment when cultured on blood agar in the vicinity of pyocyanin producing *P. aeruginosa* strains PAO1 and LES 431 (Fig. 14). It is also noteworthy that formation of the μ-oxo dimeric haem-containing pigment by *P. gingivalis* from Fe(II) haems (liberated from haemoglobin) would tie up superoxide [19]. In addition, both the μ-oxo dimer and the haematin monomer (Fe(III)PPIX.OH), which comprises 10–15% of the *P. gingivalis* pigment [19], and is the main component of the *P. intermedia* pigment [18], also possess intrinsic catalase activity which would destroy H_2_O_2_ [77]. Whilst superoxide is also potentially harmful to the bacterium, it is noteworthy that *P. gingivalis* has an intracellular superoxide dismutase [78,79] to cope with fluxes of this oxidant. In addition, in the lung it is likely that the cell-surface haem pigments of these two species would also offer a degree of protection through the ability of the ferrihaems to oxidatively inactivate pyocyanin in the presence of H_2_O_2_ [80].

In this study we showed that pyocyanin induced rapid haemoglobin oxidation over a range of concentrations found in the sputum of CF patients. For example, 100 μM pyocyanin induced an oxidation rate 40-fold that of the natural auto-oxidation, whilst the lowest concentration tested (5 μM) mediated a 5-fold increase compared to auto-oxidation. This is notable since the average level of pyocyanin production in cultures of clinical non-epidemic, non-pyocyanin over-producers and environmental isolates of *P. aeruginosa*, is around 4 μM [39]. It is therefore likely that any free oxyhaemoglobin entering the lung would become unavoidably oxidised in the presence of pyocyanin and would represent a ready pool of methaemoglobin for breakdown and haem release. In this respect, after 24 h there was a statistically significant elevation in the percentage of methaemoglobin in lung homogenates from mice given *P. gingivalis* plus pyocyanin compared to lungs of mice challenged with *P. gingivalis* only. We speculate that the presence of pyocyanin, through augmenting the natural (auto-oxidation) levels of methaemoglobin, is a means by which haem availability may be increased sufficiently to establish infection and/or to enhance the virulence of other haem-requiring species.

Here we have demonstrated that pyocyanin facilitates extraction of haem from haemoglobin by the *P. gingivalis* HmuY haemophore by rapidly oxidising oxyhaemoglobin to methaemoglobin. These findings would suggest that oxyhaemoglobin “primed” by reaction with pyocyanin is a ready and facile substrate which *P. gingivalis* may utilise to gain haem when entering the infected lung, and place these bacteria at an advantage during their early colonisation of this environment. However, in the absence of sufficient bacterial protease activity (as may be the case during the early stages of infection) it is likely that neutrophil elastase, which is abundant in the CF lung during infection [31,32], serves the role of degrading methaemoglobin for subsequent haem capture via bacterial haemophores. We note that instillation of pyocyanin into the lung can result in pathological changes [37], and here we showed that pyocyanin itself promoted a small degree of inflammatory neutrophil infiltration into the lung tissue as measured by raised lung homogenate myeloperoxidase activities, confirming the observations from other studies [33]. Importantly, however, we also found that myeloperoxidase activity was significantly increased in the presence of both *P. gingivalis* and pyocyanin, compared to that in lungs from mice challenged only with PBS, PBS plus pyocyanin, or with *P. gingivalis* only. Taken together, our findings also point to the likelihood that neutrophils play a significant, yet inadvertent, part in the haem acquisition process by invading bacteria, especially during co-colonisation with pyocyanin-producing *P. aeruginosa* strains. In addition, the combination of neutrophil elastase and pyocyanin may be a crucial factor for BPAs in haem procurement at stage before they can liberate sufficient haem through their own acquisition systems. In contrast, however, we found that elastase from *P. aeruginosa* was inefficient in degrading methaemoglobin formed by the action of pyocyanin compared to K-gingipain, which may advantage *P. gingivalis* in the competition for haem.

In this study we employed a mouse model to evaluate in isolation the effects of pyocyanin on mono-infection of the lung with *P. gingivalis*. This avoided any compounding pathological complications, which would arise from the presence of *P. aeruginosa* as a source of pyocyanin in a co-infection. Interestingly, the numbers of viable *P. gingivalis* cells in mouse lungs after inoculation of *P. gingivalis* or *P. gingivalis* plus pyocyanin were similar, indicating that the increased mortality of mice in the presence of both *P. gingivalis* and pyocyanin may not have been due to any increase in bacterial load but due to an increase in virulence properties. Indeed, we observed that R-gingipain activity was statistically significantly elevated in the lungs of mice challenged with both *P. gingivalis* plus pyocyanin compared to those receiving only *P. gingivalis*. It is known that growth of *P. gingivalis* under haem excess enhances virulence in mouse pathogenicity models [15], and that this increased pathogenicity correlates with increased cellular R-gingipain activity [16,60,61]. Minas *et al*. [81] have shown that growth of *P. gingivalis* in the chemostat with haemoglobin as substrate induces high levels of R-gingipain activity. They also have shown that although haem availability brings about increased biomass, it also induces greater total R-gingipain activity at concentrations beyond which it ceases to exert a growth rate enhancing effect [82]. These effects may explain the observations of similar bacterial numbers but the increased R-gingipain levels in the lungs of mice challenged with *P. gingivalis* plus pyocyanin compared to those with *P. gingivalis* alone. Thus, the excess of free haem in the lungs of mice co-inoculated with *P. gingivalis* and pyocyanin would stimulate gingipain expression which in turn would enhance neutrophil influx and activation, contributing to increased severity of infection. Moreover, we also found that pyocyanin had no effect on the growth of *P. gingivalis in vitro* when added to the bacteria either at the start of the incubation or during the lag phase of growth. Pyocyanin also had no inhibitory effect on either cell-associated or extracellular R-gingipain activity throughout the growth cycle. These data demonstrate that the presence of pyocyanin in the lung may not abrogate the activity of one of the major virulence factors of this organism, *i.e.*, gingipain proteases.

The ecological inter-relationships between anaerobes, including the haem-requiring BPAs and *P. aeruginosa* in the infected lung is not clear. However, it is known that *P. aeruginosa* also has a metabolic requirement for haem and iron [83,84], which the organism would need to establish an infection in an environment where the haem availability may otherwise be restricted by the host *via* sequestration by serum albumin and haemopexin. In the context of haem and aerobiosis, Diaz and Rogers [85] have shown that *P. gingivalis* can grow not only under strictly anaerobic conditions, but also in the presence of 6% v/v O_2._ This ability to survive in O_2_ is linked to expression of NADH oxidase, NADH peroxidase and superoxide dismutase (SOD) [85] and the importance of this anti-oxidant system is highlighted by the observation that SOD-negative mutants of *P. gingivalis* do not survive exposure to oxygen [86]. It is also significant to note that *P. gingivalis* can tolerate up to 10% oxygen in the presence of haem [85]. These physiological adaptations would permit *P. gingivalis* to co-exist in the micro-aerobic conditions (as in the CF lung), which *P. aeruginosa* can also tolerate [12,87–89].

It is generally accepted that micro-aerobic conditions prevail in the CF lung as a result of oxygen depletion due to the metabolism of aerobic bacteria and to the presence of the viscous mucus secretions which limit gas exchange and oxygenation. In this context it is important to note that Minetti *et al*. [42] demonstrated that haemoglobin oxidation by phenazine is actually increased at reduced O_2_ levels, and so pyocyanin-mediated methaemoglobin formation could proceed in the CF lung where there may be both full oxygenation and also in local microenvironments where other co-colonising species and *P. aeruginosa* [63] may induce micro-aerobic conditions. It is also noteworthy that in the *Drosophila* model of polymicrobial infection with other bacterial species of oropharyngeal origin can increase gene expression of a number of *P. aeruginosa* virulence-related genes including *phzA1*, which encodes for a protein involved in phenazine biosynthesis [90].

The mechanisms through which haem becomes available during lung infections have not been previously investigated in great detail. However, results presented here have revealed that pyocyanin rapidly oxidises oxyhaemoglobin to methaemoglobin and that this is a facile substrate for the *P. gingivalis* Kgp and the HmuY haemophore, which can extract haem from it. The presence of pyocyanin also augmented the breakdown of oxyhaemoglobin and release of haem by neutrophil elastase, which is considered to be most abundant host protease present in the CF lung during bacterial infection [30,31]. Thus, our above observations are of significance as they indicate that free haem levels may also be elevated inadvertantly by the host in response to infection by *P. aeruginosa*.

Data published by others, along with our results presented here demonstrate that cooperation exists between the respiratory pathogens and certain oral bacteria, such as *P. gingivalis*, thus enhancing their virulence. For example, it has been found that *P. gingivalis* may increase invasion into, and transiently suppress *P. aeruginosa*-induced apoptosis of respiratory epithelial cells, which may facilitate intracellular pathogen proliferation and dissemination of infection [91,92]. In addition, mixed invasion with both of these bacterial species triggers more epithelial cell death. Whilst this may be advantageous to the host in that it may contribute to elimination of infected cells, it also causes tissue destruction. Several studies also support the importance of encapsulated anaerobic bacteria in respiratory infections [93]. Moreover, when relatively non-encapsulated isolates of *Porphyromonas* strains were mixed with *P. aeruginosa*, enhanced virulence of the latter was observed. Confirming our hypothesis of synergy between *P. aeruginosa* and *P. gingivalis*, it has been also very recently demonstrated that the relative loads of *P. gingivalis* compared to *P. aeruginosa* were higher in tracheal aspirate samples than in the corresponding periodontal pocket samples [5].

In summary, although pyocyanin *per se*, is known to bring about pathological changes in the lungs of experimental animals when administered repeatedly in high doses [37], we have demonstrated for the first time that it has a significant effect on haem availability by increasing the level of methaemoglobin in the lung during pulmonary challenge with *P. gingivalis*. In addition, the presence of pyocyanin results in increased expression of R-gingipain protease activity, a major *P. gingivalis* virulence factor. Our findings also show that the paradigm of haem acquisition displayed by the BPAs may extend to co-infections of these bacteria with *P. aeruginosa* and offer a biochemical mechanism for the ability of BPAs to obtain sufficient haem in the infected lung. An increased abundance of iron protoporphyrin IX may also enable BPAs to endure the micro-aerobic environment and to contribute to the growth and virulence of other haem-requiring bacterial species and thus help to shape the microbiological profile in the infected lung. Our study demonstrates that anaerobic pathogens, such as *P. gingivalis*, are worthy of further study in the context of a variety of pulmonary infections, including those of the CF lung, as well as in the context of their wider role of the lung microbiome. Because knowledge regarding the association of anaerobic bacteria with lung diseases, and whether or how they should be treated is limited, our study provides new data which should be treated as the basis for future clinical experiments. It is thus likely that chemotherapeutic methods which restrict availability of virulence regulatory factors, such as haem, may need to be developed for treating lung infections, mostly recurring lung infections in CF patients.

## Materials and Methods

### Isolation and purification of HmuY

*P. gingivalis* apoHmuY lacking the first 25 residues (NCBI accession number CAM 31898) was expressed using a pHmuY11 plasmid and *Escherichia coli* ER2566 cells (New England Biolabs) and purified from a soluble fraction of the *E. coli* cell lysate as described previously [25]. As the soluble protein released from the cell membrane, the purified HmuY lacked the signal peptide and first five amino acid residues (CGKKK) of the nascent secreted protein [25,27].

### Haemoglobin preparations

Oxyhaemoglobin for use in oxidation rate measurements and protease degradation experiments was prepared from fresh horse erythrocytes as described previously [53] and stored at -80°C in 0.14 M NaCl, 0.1 M Tris-HCl, pH 7.5 until required. Methaemoglobin was formed by incubation of 100 μM oxyhaemoglobin with a 4-fold molar excess of NaNO_2_ for 1 h at 37°C [22,23] or with *P. aeruginosa* pyocyanin (Sigma-Aldrich; P0046) at concentrations between 5 and 100 μM in 0.14 M NaCl, 0.1 M Tris-HCl, pH 7.5.

### Rates of oxyhaemoglobin oxidation

The rates of oxyhaemoglobin (HbO_2_) oxidation were also followed quantitatively using plots of log([HbO_2_]_*t*_/[HbO_2_]_*t0*_) against time *t*, where the ratio of HbO_2_ concentration after time *t* to that at time zero (*t*_*0*_) was monitored by the absorbance changes of the α band (576 nm) of oxyhaemoglobin [24,46]. The relative rates of oxidation were calculated by linear regression using GraphPad Prism.

### Determination of the concentration of methaemoglobin

The concentrations of methaemoglobin in oxyhaemoglobin preparations were calculated according to the method of Winterbourne *et al*. [45] using the A_576_ and A_630_ values as described previously [22].

### Assay for total haem and protein in lung tissues

Parallel aliquots of lung homogenates were centrifuged as above to pellet insoluble tissue residues plus the biomass (in the case of the infected samples). Portions of this were solubilised in 0.1 M NaOH for 15 min in a sonication bath. The samples were then centrifuged at 18,000×*g* for 5 min and any lipid floating on the top of the supernatant carefully removed. The soluble fraction thus obtained was then assayed for total protein assay using the bicinchoninic acid (BCA) method, and for haem using the method of Pandey *et al*. [94]. The assays were calibrated against haemin chloride (Sigma-Aldrich) and bovine serum albumin.

### UV-visible spectroscopy

UV-visible spectra were recorded with a Ultrospec 2000 spectrophotometer (Biochrom Ltd) using 1-cm pathlength cuvettes.

### Human neutrophil elastase

Neutrophil elastase (BioCentrum Ltd., Krakow, Poland; E-001) had a specific activity of 20.8 U/mg against the synthetic substrate N-methoxysuccinyl-Ala-Ala-Pro-Val-*p*-nitroanilide (MeOSuc-Ala-Ala-Pro-Val-pNA). Determination of the potential inhibitory effect of pyocyanin towards elastase (25 nM) was carried out using 250 μM MeOSuc-Ala-Ala-Pro-Val-pNA (SigmaAldrich; M6475) and Azocoll (Calbiochem; final concentration 7.5 mg/ml) in 0.14 M NaCl, 0.05 M Tris-HCl, 5 mM EDTA and 0.05% Tween-20, pH 7.5 at 37°C.

### K-gingipain (Kgp) purification

Kgp was purified from the culture medium of *P. gingivalis* strain HG66 as described previously using gel filtration and arginine–Sepharose chromatography [95].

### Pseudomonas elastase

*Pseudomonas* elastase was purchased from Calbiochem (product number 324676)

### Polyacrylamide gel electrophoresis (PAGE)

PAGE in the presence of SDS was carried out using the buffer system of Laemmli [96]. To demonstrate haemoglobin proteolysis, samples were solubilised in application buffer for 1 h at 37°C without dithiothreitol. Where appropriate for native PAGE, urea and SDS were omitted from the separating gel, and the samples were also solubilised for 1 h at 37°C in application buffer but without SDS, urea and dithiothreitol. The gels were firstly stained for the presence of haem using tetramethylbenzidine/H_2_O_2_ (TMB-H_2_O_2_), and then counter-stained with Coomassie Brilliant Blue R-250 (CBB) to visualise the protein as previously described [28].

### Bacterial strains and cultures

*P. gingivalis* strains W83 and W50 were used in this study. Bacteria were grown under anaerobic conditions (90% N_2_, 5% CO_2_, 5% H_2_) on blood (5% v/v sheep blood) agar plates or in liquid Schaedler broth (BTL, Lodz, Poland) supplemented with haemin (5 μg/ml; SigmaAldrich), L-cysteine (50 μg/ml; SigmaAldrich), menadione (0.5 μg/ml; SigmaAldrich). Bacteria from an overnight culture were centrifuged and the bacterial pellet was washed three times with phosphate–buffered saline (0.14 M NaCl, 0.0026 M KCl, 0.01 M Na_2_HPO_4_, 0.002 M KH_2_PO_4_), pH 7.4 (PBS), and re-suspended in PBS. Bacterial cell counts were standardized to an optical density of 1.0 at 600 nm (corresponding to 1×10^9^ CFU ml^-1^).

### Experimental animals

Specific pathogen-free (SPF) female C57/BL/6 mice, 10–12 weeks of age, were purchased from Harlan Laboratories (Udine, Italy). Mice were housed in positively-ventilated micro-isolator cages, were fed a standard laboratory diet, located in a room with laminar, high efficiency particle accumulation-filtered air within the animal care facility at the Jagiellonian University (Krakow, Poland). Control and bacterially-infected mice were housed in separate cages.

### Ethical approval

All animal studies were performed in accordance with the protocols laid down by the Institutional Animal Care and Use I Regional Ethics Committee on Animal Experimentation, Krakow, Poland. This specific animal study was approved by the above ethics committee (Decision No. 94/2009).

### Lung infection model in C57/BL/6 mice

**Fig 15:**
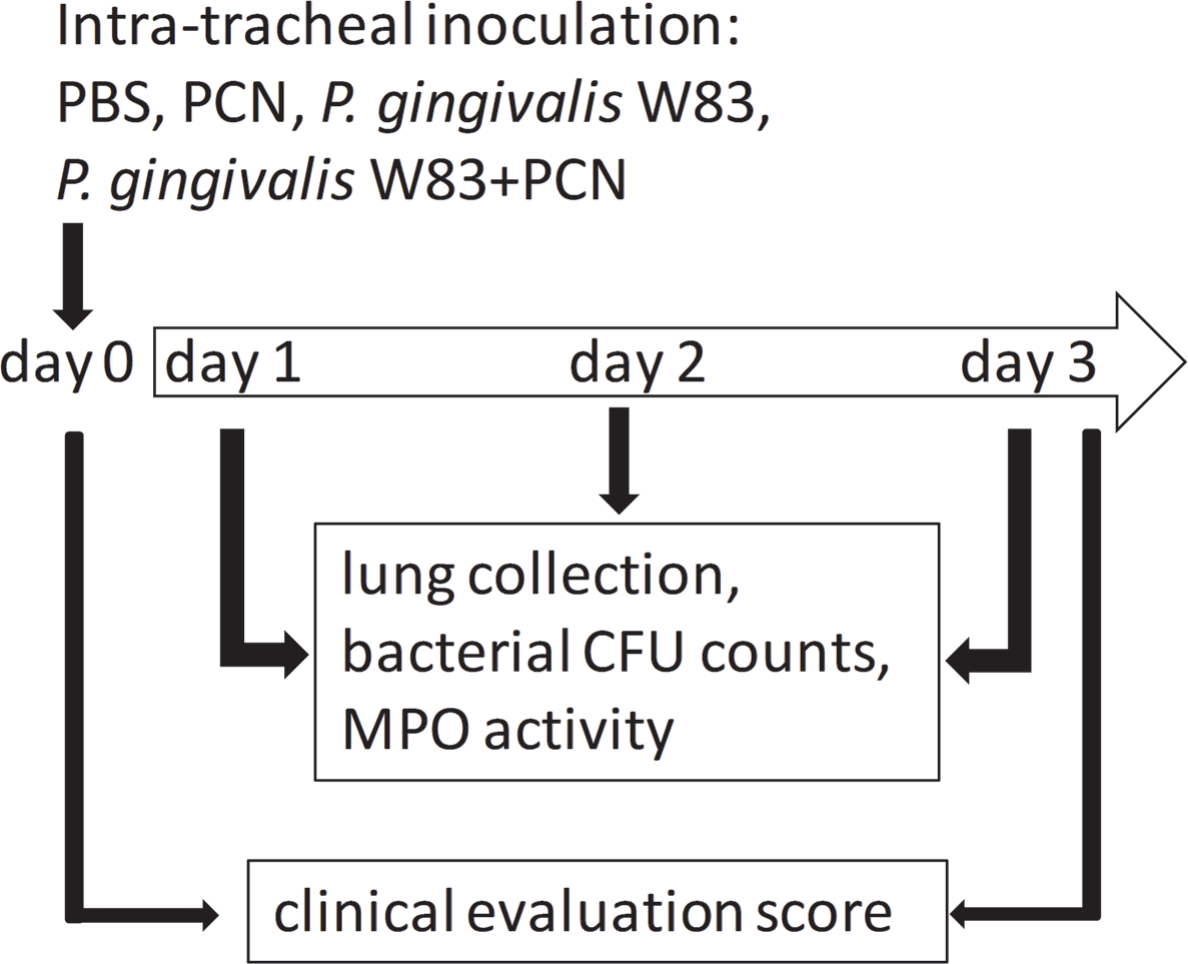
Flow diagram of the experimental design and sampling protocol for the mouse lung infection model.

The mouse lung infection model of Nemec *et al*. [44] was followed, employing an infectious dose of *P. gingivalis* of 3×10^9^ CFU. In these experiments we evaluated the effect of only one variable (*i.e.*, pyocyanin) on the virulence of *P. gingivalis* in the absence of *P. aeruginosa*, which would otherwise introduce other confounding pathological variables. For this we used an intra-tracheal challenge of 40 μg pyocyanin, a dose in line with that used in the study of Reszka *et al*. [80]. Mice were exposed to pyocyanin and/or to viable *P. gingivalis* W83 cells according to the following experimental protocols. To minimize any pain or distress during these experimental procedures, the C57/BL/6 mice were firstly anesthetized by intraperitoneal injection of ketamine (22 mg/kg; VetaKetam, Vet-Agro, Poland), and xylazine (2 mg/kg; Sedasin, Biowet) and ointment (Puralube Vet; Pharmaderm) applied to the eyes to prevent them from drying. The lungs of the animals were then challenged non-surgically by intra-tracheal administration of inocula (50 μl) containing 3×10^9^ CFU of viable *P. gingivalis* W83 cells with or without 40 μg of pyocyanin (PCN; Sigma-Aldrich) in PBS. To minimize any damage to the airways, this was done using a blunt-ended 21-gauge silicone cannula needle which was applied to the back of the tongue above the tracheal opening. The animals were then maintained head upper most at a 45° angle until fully recovered from the anesthesia. Successful delivery of the PBS or bacteria with or without pyocyanin to the lungs was manifested in a choking reflex by the mouse immediately after instillation, followed by rapid breathing. Control mice were inoculated with 50 μl PBS or 50 μl PBS containing 40 μg pyocyanin. Three separate sets of *in vivo* experiments were conducted employing groups of 10 mice. All experimental procedures are summarized in Fig. 15.

### CFU assay

On days 1, 2 and 3, mice were sacrificed by cervical dislocation and the lungs were immediately removed from the animals, weighed for purposes of standardization of the CFU and myeloperoxidase assays, homogenized in 1 ml of PBS using TissueLyser (Qiagen), and the homogenates 10-fold serially diluted. All samples (100 μl each) were plated on blood agar plates and cultured anaerobically for 7 days at 37°C, after which time visible colonies of *P. gingivalis* were counted to obtain the total viable cell numbers.

### Determination of haemoglobin concentrations in mouse lung homogenates

Mouse lung homogenates as prepared above (0.3 ml) were diluted with 0.7 ml PBS, pH 7.4, and then centrifuged at 18,000×*g* for 20 min to pellet tissue residues. The clear supernatant fractions thus formed were carefully removed and UV-visible spectra recorded in 1-cm path length cuvettes. After subtraction of the buffer background, the absorbance values at 576 and 630 nm were used to calculate the concentrations of both oxyhaemoglobin and methaemoglobin by multicomponent analysis using the equations described previously [22].

### Clinical evaluation scores

Scoring was made according to the clinical responses of C57/BL/6 mice to intra-tracheal inoculation with *P. gingivalis* W83 or *P. gingivalis* plus pyocyanin, and with PBS, or PBS plus pyocyanin. The clinical status of the mice was assessed during three days after challenge and included visual examination for mortality, respiratory failure, loss of body weight, feeding disruption, ruffled fur, loss of activity, ataxia and tremor. The clinical score from 0 to 3 was based on degree of physical symptoms (0, no sign; 1, mild; 2, moderate; 3, severe). All animals were monitored hourly over the experimental period. Any animals displaying severe or chronic pain or distress, *e.g.*, immediate inactivity, breathing troubles and/or bleeding from the nose and mouth after inoculation of bacteria, pyocyanin or PBS were humanely euthanized. This was done by firstly anaesthetizing the animals as above by intraperitoneal injection with ketamine (22mg/kg) and xylazine (2mg/kg), followed by cervical dislocation.

### Myeloperoxidase assay

Neutrophil influx into the lung tissue was analyzed by assaying for the enzymatic activity of myeloperoxidase, a marker for neutrophil accumulation [59]. Briefly, the lung tissues were weighed and homogenized in PBS, pH 7.4, containing 5 mg/ml hexadecyltrimethylammonium bromide, to produce 0.05% v/v homogenates. The homogenates were centrifuged at 40,000×*g* for 15 min and 10 μl of the supernatants were mixed with 240 μl dianisidine reagent comprising 16.7 mg dianisidine, 90 ml of distilled water, 10 ml PBS, and 50 μl of 0.29 M hydrogen peroxide. The reaction mixture was incubated for 5 min at room temperature, terminated by addition of 50 μl 2 M H_2_SO_4_, and then three absorbance readings at 30-s intervals were made using a micro-titre plate scanner at 450 nm as direct measure of myeloperoxidase activity. The activities in all samples were expressed as % of controls (lung homogenates from animals inoculated with PBS only).

### Gingipain expression in lung tissues

Homogenates of the lung tissues were prepared as described above. In order to measure gingipain proteolytic activity, 5 μl of tissue homogenate were added to 95 μl of test buffer (100 mM Tris pH 7.5, 200 mM Gly-Gly, 5 mM CaCl_2_, 20 μg/ml aprotinin, 100 μg/mL PEFABLOCK) and supplemented with 10 mM L-cysteine in a 96-well microtitre plate. After 5-min pre-incubation at 37°C, the gingipain substrate benzoyl-arginine-*p*-nitroanilide (BApNA; SigmaAldrich) was added to give a working concentration of 200 μM. The protease activity was followed by measuring the release of *p*-nitroaniline by determination of absorbance at 405 nm using a microplate reader (SpectraMax Gemini, Molecular Devices) as described previously [97].

### Effect of pyocyanin on the *P.gingivalis* growth *in vitro*

*P.gingivalis* strain W83 was grown in liquid Shaedler broth (BTL, Lodz, Poland) supplemented with haemin chloride (5 μg/mL; SigmaAldrich; product number H9039), L-cysteine (50 μg/mL; SigmaAldrich) and menadione (0.5 μg/mL; SigmaAldrich) under anaerobic conditions (90% N_2_, 5% CO_2_, and 5% H_2_). The influence of pyocyanin on the growth of *P. gingivalis* growth was examined by the adding pyocyanin (40 μg ml^-1^) to the bacterial cultures (3×10^9^ cfu ml^-1^) either at the start of the incubation or 4 h after inoculation to test the effect in the early growth phase. The bacterial growth was monitored by measuring the OD_600_. Pyocyanin was omitted in the controls. The assays were done in duplicate.

### Effect of pyocyanin on bacterial R-gingipain *in vitro*

*P. gingivalis* strain W83 (3×10^9^ cfu ml^-1^) was grown as above in the absence and presence of pyocyanin (40 μg/ml) and the cultures were sampled at the start and at 6, 12, 18, 38 h post-inoculation and the cell-associated and extracellular gingipain activity assayed using BApNA as above after centrifugation of the cultures at 18,000×*g* for 15 min. The protease assays at each time period were performed on six replicate samples.

### Growth of *P. gingivalis* in the presence of secreted products of *P. aeruginosa*

*P. aeruginosa* strains PAO1 and LES 431 were grown overnight at 37°C as single large colonies on Columbia horse blood agar plates, after which time pyocyanin was observed as a halo around the growth. *P. gingivalis* W50 was maintained anaerobically on horse blood agar and then streaked radially at a short distance from, but not touching, the *P. aerugnosa* colonies and the plates incubated anaerobically for 4 days. Pyocyanin producing *P. aeruginosa* strains PAO1 and LES 431 were kindly provided by Professor Craig Winstanley, Institute of Infection and Global health, the University of Liverpool.

### Statistical analysis

All the data were reported as mean ± SD. To determine the significance of the results obtained from the *in vivo* experiments, comparisons between groups were made using the Mann-Whitney U test. Two-way ANOVA was employed for comparison of multiple groups. Results for the haem and methaemoglobin contents in mouse lungs were analyzed using Kruskal-Wallis one-way analysis of variance. The statistical significance of the gingipain protease expression data was tested using the Student's *t*-test with Welch’s correction. Differences between the numbers of viable *P. gingivalis* cells were analysed using Student's *t*-tests with equal (bacterial number after 16 h) or unequal (bacterial number after 24 h) variances. Values of *P* ≤ 0.05 were considered to be statistically significant.
