# Correction: Pyocycanin, a Contributory Factor in Haem Acquisition and Virulence Enhancement of *Porphyromonas gingivalis* in the Lung

**DOI:** 10.1371/journal.pone.0148008

**Published:** 2016-01-25

**Authors:** Malgorzata Benedyk, Dominic P. Byrne, Izabela Glowczyk, Jan Potempa, Mariusz Olczak, Teresa Olczak, John W. Smalley

The word “Pyocyanin” is misspelled in the article title. The correct title is: Pyocyanin, a Contributory Factor in Haem Acquisition and Virulence Enhancement of *Porphyromonas gingivalis* in the Lung. The correct citation is: Benedyk M, Byrne DP, Glowczyk I, Potempa J, Olczak M, Olczak T, et al. (2015) Pyocyanin, a Contributory Factor in Haem Acquisition and Virulence Enhancement of *Porphyromonas gingivalis* in the Lung. PLoS ONE 10(2): e0118319. doi:10.1371/journal.pone.0118319

The following information is missing from the Funding section: The Faculty of Biochemistry, Biophysics and Biotechnology of Jagiellonian University is a partner of the Leading National Research Center (KNOW) supported by the Ministry of Science and Higher Education.

## References

[1] Benedyk M, Byrne DP, Glowczyk I, Potempa J, Olczak M, Olczak T, et al (2015) Pyocycanin, a Contributory Factor in Haem Acquisition and Virulence Enhancement of *Porphyromonas gingivalis* in the Lung. PLoS ONE 10(2): e0118319 doi: 10.1371/journal.pone.0118319 25706529 10.1371/journal.pone.0118319 PMC4338185

